# Evaluating the environmental impact of cleaning the North Pacific Garbage Patch

**DOI:** 10.1038/s41598-025-00619-w

**Published:** 2025-05-27

**Authors:** Matthias Egger, Andy M. Booth, Thijs Bosker, Gert Everaert, Samantha L. Garrard, Vilma Havas, Helga S. Huntley, Albert A. Koelmans, Karin Kvale, Laurent Lebreton, Helge Niemann, Qiaotong Pang, Maira Proietti, Peter Puskic, Camille Richon, Sarah-Jeanne Royer, Matthew S. Savoca, Arjen Tjallema, Marjolein van Vulpen, Yanxu Zhang, Ziman Zhang, Denise M. Mitrano

**Affiliations:** 1 The Ocean Cleanup, Rotterdam, The Netherlands; 2 Empaqtify, St. Gallen, Switzerland; 3 SINTEF Ocean, Trondheim, Norway; 4 Leiden University, Leiden, The Netherlands; 5 Flanders Marine Institute, Ostend, Belgium; 6 Plymouth Marine Laboratory, Plymouth, UK; 7 Salt Lofoten, Arendal, Norway; 8 Aalborg University, Aalborg, Denmark; 9 Rowan University, Glassboro, NJ USA; 10 Aquatic Ecology and Water Quality Management Group, Wageningen University, Wageningen, The Netherlands; 11 GNS Science, Avalon, Lower Hutt, New Zealand; 12 Aotearoa Blue Ocean Research, Lower Hutt, New Zealand; 13 The Modelling House Limited, Raglan, New Zealand; 14 NIOZ Royal Netherlands Institute for Sea Research, ‘t Horntje (Texel), The Netherlands; 15 Department of Earth Sciences, Faculty of Geosciences, Utrecht University, Utrecht, The Netherlands; 16 Joint International Research Laboratory of Atmospheric and Earth System Sciences, School of Atmospheric Sciences, Nanjing University, Nanjing, China; 17 Universidade Federal do Rio Grande (FURG), Rio Grande, Brazil; 18 Institute for Marine and Antarctic Studies, University of Tasmania, Hobart, Australia; 19 Center for Marine Socioecology, Hobart, Australia; 20 CNRS, Ifremer, IRD, Laboratoire des Sciences de l’Environnement Marin (LEMAR), IUEM, University Brest, Brest, France; 21 Hopkins Marine Station, Stanford University, Pacific Grove, CA USA; 22 California Marine Sanctuary Foundation, Monterey, CA USA; 23 Department of Earth and Environmental Sciences, Tulane University, New Orleans, LA USA; 24 Swiss Federal Institute of Technology (ETH) Zurich, Zurich, Switzerland

**Keywords:** Marine debris, Ocean plastic pollution, Legacy oceanic plastic pollution, Open-ocean cleanup, Net environmental benefit analysis, Environmental impact, Marine biology

## Abstract

Cleanup of existing plastic pollution is crucial to mitigate its impact on marine ecosystems, but such efforts must ensure benefits outweigh potential environmental damage caused by the cleanup. Here, we present an impact assessment framework and apply it to evaluate whether cleaning the North Pacific Garbage Patch (NPGP) benefits marine life and carbon cycling, using The Ocean Cleanup as a case study. Our findings indicate that marine life is more vulnerable to plastic pollution than to macroplastic cleanup, with average vulnerability scores (1 = low, 3 = high) of 2.3 for macroplastics, 1.9 for microplastics, and 1.8 for cleanup, suggesting a net positive impact. An 80% cleanup could reduce macroplastic concentrations to within reported safe levels for marine mammals and sea turtles. Estimated cleanup-related carbon emissions [0.4–2.9 million metric tons (Mt) in total] are significantly lower than potential long-term microplastics impacts on ocean carbon sequestration (15–30 Mt C per year). However, uncertainties remain regarding effects on air-sea carbon exchange. Our framework serves as a critical tool for assessing trade-offs between plastic pollution and remediation impacts. It demonstrates the environmental net benefits of the proposed NPGP cleanup and can be adapted to similarly evaluate other remediation plans.

## Introduction

Increasing scientific evidence of potential detrimental consequences for marine life—such as ingestion, entanglement, habitat disruption, and toxic chemical exposure^1,2^—has led to calls for urgent action to address ocean plastic pollution. Globally, the issue is addressed via a range of legislative approaches, including the phase-out of single-use plastics^3^, the introduction of extended producer responsibility schemes, development of alternative biodegradable substitutes, regulation of primary microplastics^4^, and implementation of trade restrictions on plastic waste transport^5^. National and regional actions, however, fail to fully address the severity and global scale of plastic pollution, and allow for problem transfer between regions^6^. Therefore, international negotiations are underway to develop a Global Plastics Treaty, a legally binding instrument on plastic pollution, including in the marine environment^7^. While prevention of further pollution is critical, measures to reduce future plastic emissions do not address impacts of plastics that have already accumulated in the environment^8^. Without any action, this legacy plastic pollution will continue to reside in and impact ecosystems, including the marine environment, for decades or centuries to come^9,10^.

**Fig. 1:**
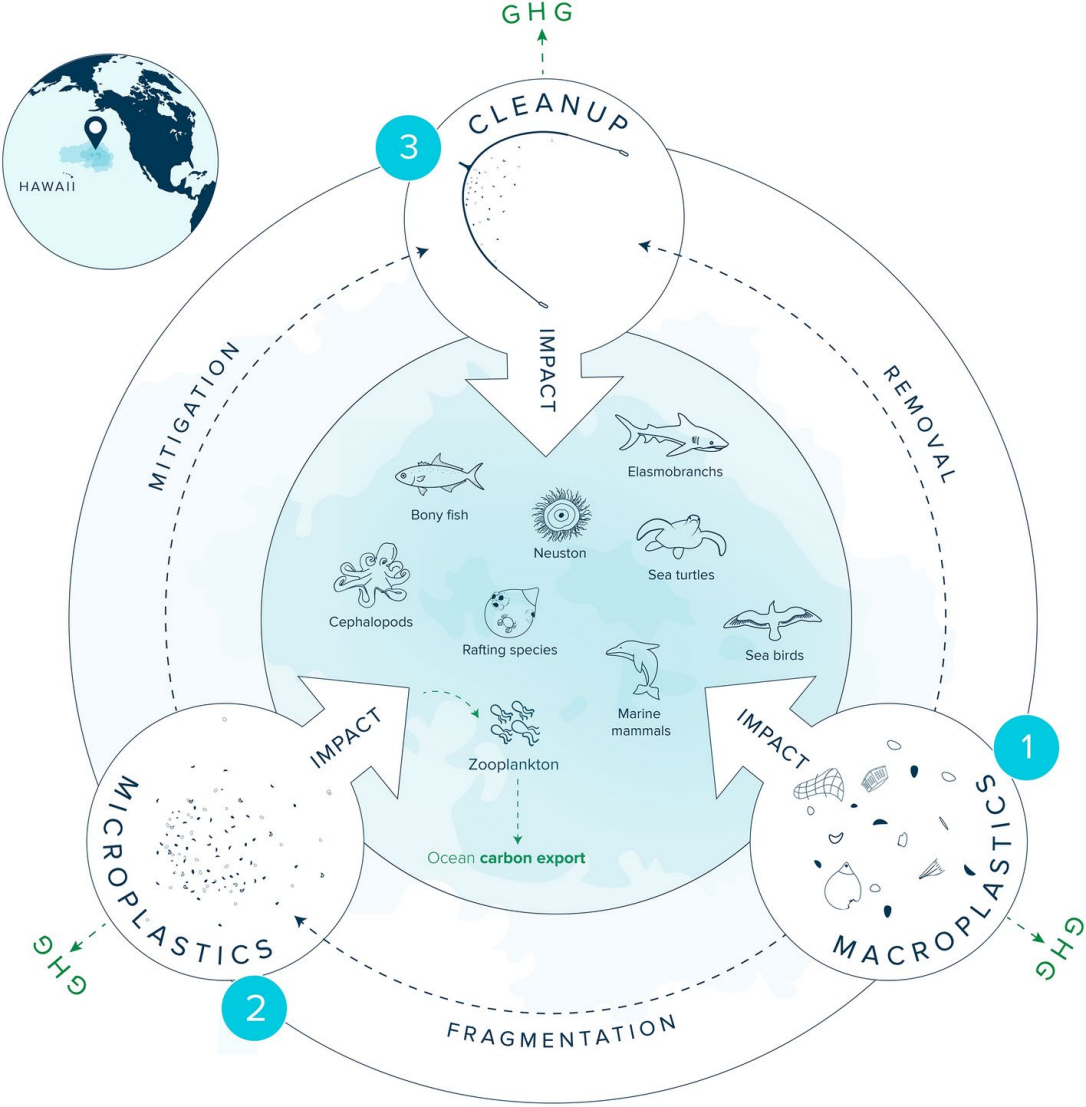
Schematic of the impact assessment framework developed in this study to evaluate the net environmental impact of cleaning the North Pacific Garbage Patch (NPGP). Impacts of three anthropogenic stressors (1) macroplastics (> 5 mm), (2) microplastics (1 µm–5 mm), and (3) cleanup on nine ecological guilds (zooplankton, neuston, bony fish, elasmobranchs, sea turtles, seabirds, marine mammals, cephalopods and rafting species) were assessed to evaluate impacts on marine life. Impacts on carbon cycling were assessed by estimating greenhouse gas (GHG) emissions from plastics afloat in the NPGP, vessel emissions during cleanup, and potential microplastic impacts on ocean carbon export in the NPGP.

More than half of the plastic mass produced annually consists of polymer types with a density lower than seawater^11,12^. After entering the marine environment, these positively buoyant plastics either beach back to land relatively quickly^13–15^, reside in coastal waters^9^, or are transported offshore where they can accumulate in subtropical oceanic gyres^16–21^. The highest concentrations of plastics in the open ocean have been recorded in the North Pacific subtropical gyre, located between California and Hawai’i^17–19^, also known as the North Pacific Garbage Patch (NPGP, Fig. 1). Plastics in the NPGP have been shown to persist for at least decades^9,14,22^, and to mainly originate from fishing^20,21^.

Abandoned, lost, or discarded fishing gear (ALDFG) is particularly harmful to marine life due to continued ghost fishing^23^. Consequently, the NPGP is a hotspot for plastic entanglement^24^ and a high plastic exposure risk area for threatened species^25^. Because of its oligotrophic nature and relatively high microplastic concentrations, the NPGP is particularly sensitive to microplastics impacts^26–28^. Additionally, plastics afloat in the NPGP can contain high levels of chemical additives^29^ and harbor a variety of coastal species, including potentially invasive (i.e., coastal) and pathogenic species^30–33^.

A challenging aspect of ocean plastic pollution is the fragmentation of larger plastic items into smaller plastic fragments^34–36^. These secondary micro- and nanoplastics can then be dispersed throughout the water column^37–40^. Even with an immediate elimination of emissions, the number of micro- and nanoplastic particles in the ocean is likely to increase significantly due to the slow fragmentation of larger legacy plastics into smaller pieces^8,10,14^. Legacy plastic pollution constitutes a toxicity debt, with many more years of decay and release of toxic chemicals and micro- and nanoplastics, and may be responsible for much of the present and future ecological and environmental damage associated with ocean plastic pollution^10,41–43^.

Effective mitigation of plastic pollution impacts therefore requires a drastic reduction of plastic emissions into the ocean in combination with removal of legacy plastic pollution, including in areas beyond national jurisdictions^8,10,14,44^. However, offshore cleanup efforts have been met with criticism due to the potential for environmental damage such as the possible entrapment of marine life, greenhouse gas (GHG) emissions into the atmosphere during cleanup activities, and the potential for diverting attention and resources from upstream interventions^45–47^. This highlights the need to quantify the environmental benefits of cleanups to assess whether they outweigh potential environmental costs^46,48,49^.

The current draft of the global Treaty to End Plastic Pollution includes provisions for the environmentally sound removal of existing plastic pollution, including in areas beyond national jurisdiction^50,51^. However, the definition of ‘environmentally sound’ and the criteria for assessing it remain unclear. Our work provides a practical example of what such an assessment could look like. We offer a tool to guide cleanup activities and ensure their net benefit to the environment. Specifically, we propose a plastic pollution remediation impact assessment strategy to compare the following competing management actions: (1) leaving the macroplastic debris in the ocean and (2) removing it by cleanup actions. To do so, we applied a net environmental benefit analysis (NEBA) approach^52,53^. A NEBA compares and ranks multiple management alternatives according to the net environmental benefits associated with each, where net environmental benefits are defined as gains in the value of environmental services or other ecological properties attained by remediation or ecological restoration minus the value of adverse environmental impacts caused by those actions^52,53^.

Our NEBA framework developed for the NPGP was applied to two categories, namely marine life and carbon cycling, which we considered separately. For each category, impacts associated with three separate anthropogenic stressors were assessed: (1) macroplastic pollution (here defined as > 5 mm), (2) microplastic pollution (1 µm–5 mm), and (3) cleanup activities (Fig. 1). Although cleanups focus on macroplastic pollution only, microplastics have been included in this assessment as the removal of macroplastic pollution already accumulated in the ocean ultimately avoids the generation of secondary microplastics^10,14,34^. Nanoplastics (< 1 µm) were excluded as their concentrations, transport, and impacts (including leaching of additives and impurities) in marine ecosystems are currently largely unknown^41,54–59^.

**Fig. 2:**
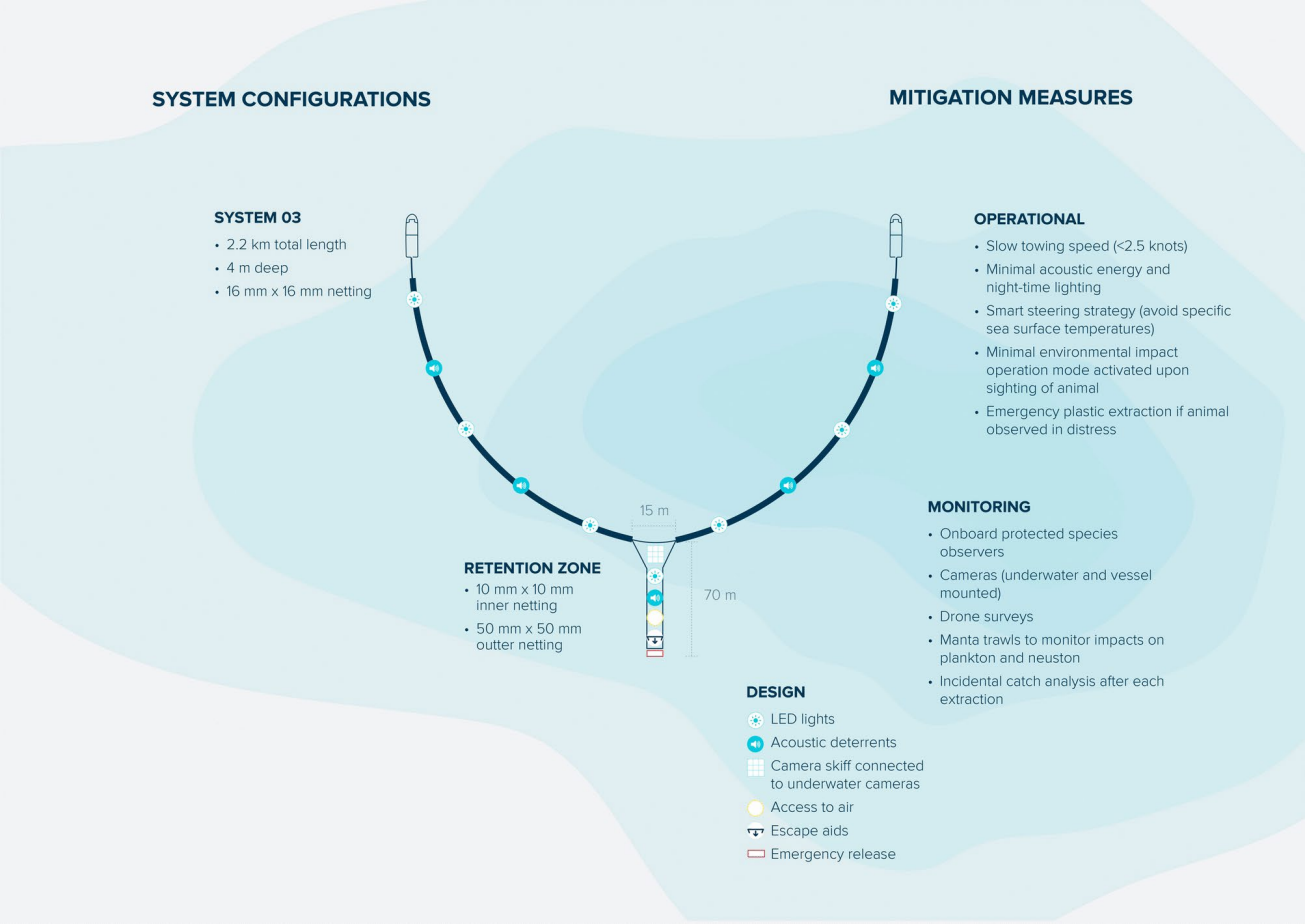
Schematic overview of The Ocean Cleanup’s System 03 and associated mitigation measures (i.e., operations, monitoring, and design). System 03 consists of a retention zone attached to a floating barrier and is towed between two slow-moving (< 2.5 knots) vessels. Using monitoring data, numerical modelling of plastic dispersal, and artificial intelligence, the system is steered towards plastic hotspot areas within the NPGP. Floating plastic debris is guided by the wings towards the retention zone. Once the retention zone is ready to be emptied (on average after ~ 4 days), it is pulled onto the vessel and the plastic is emptied on deck for sorting and packing. Subsequently, the retention zone is returned to the water, and the process begins again.

This framework was then applied to evaluate whether cleaning up the NPGP could have a net positive impact on marine life and carbon cycling in the region, based on the technology developed by the not-for-profit organization The Ocean Cleanup (Fig. 2). We emphasize that this study represents a first attempt to assess relative plastic pollution and cleanup impacts in the NPGP, which should be periodically re-assessed and improved as new data become available.

## Results

### Impacts on marine life

**Fig. 3:**
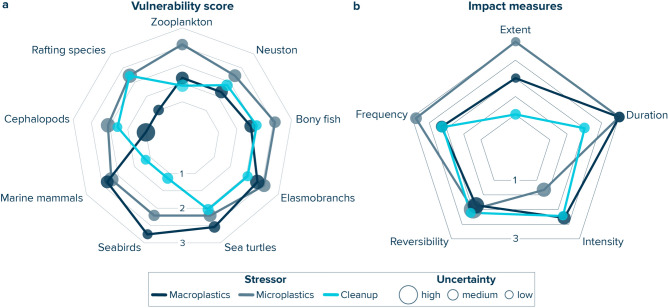
(**a**) Vulnerability scores of nine ecological guilds towards macroplastic (> 5 mm; dark blue), microplastics (1 µm–5 mm; grey) and cleanup (light blue) impacts in the North Pacific Garbage Patch. A value of one and three represents a low and high vulnerability, respectively. (**b**) Average values for the extent, duration, intensity, reversibility, and frequency of macroplastics (dark blue), microplastics (grey), and cleanup (light blue) impacts across all nine ecological guilds considered in this study. The size of the markers is scaled to the respective uncertainty scores, with larger size corresponding to larger uncertainty. Note that all values are provided in Tables S2 and S3.

By scoring the extent, duration, intensity, reversibility and frequency of impacts (Tables S1 and S2), we assessed the vulnerability of marine life in the NPGP to plastic pollution and cleanup activities (on a scale of 1 (low) to 3 (high)). Our results reveal average ecological vulnerability scores (± 1 standard deviation) for macroplastics, microplastics, and cleanup of 1.9 (± 0.6), 2.3 (± 0.2), and 1.8 (± 0.4), respectively (Table S3). We find a significant difference between vulnerability scores for microplastics and cleanup (Wilcoxon–Mann–Whitney test: p = 0.002), but no statistically significant differences between vulnerability scores of macroplastics vs. microplastics (p = 0.302) and macroplastics vs cleanup (p = 0.626). Zooplankton, marine mammals, seabirds, sea turtles, and elasmobranchs all exhibit lower vulnerability scores for cleanup compared to scores for macro- and microplastic pollution (Fig. 3a). Neuston, bony fish, cephalopods, and rafting species have lower vulnerabilities towards macroplastics compared to cleanup impacts, but higher vulnerabilities to microplastics. A detailed description of the impact scoring for each ecological guild is presented in the Supplementary Information.

Based on our assessment, impacts from macroplastics and cleanup typically refer to entanglement and are relatively high in intensity, difficult to reverse, and occur occasionally. However, cleanup impacts are assumed to be limited in their geographical extent and duration, while impacts of macroplastics in the NPGP are considered to be long-term (i.e., > 10 years) and extend beyond the NPGP, particularly onto the Hawaiian archipelago due to macroplastics escaping from the NPGP as a result of oceanographic transport (Fig. 3b, Table S2). Microplastic impacts, on the other hand, are estimated to be low in intensity (mostly from ingestion), but abundant and extending beyond the ocean surface due to their vertical transport away from the surface ocean into the ocean water column (Fig. 3b). We further identify higher confidence for impacts related to cleanup compared to plastic pollution impacts, particularly compared to microplastics, with an average (± 1 standard deviation) uncertainty score (ranging from 0.2 (low) to 1 (high)) of 0.5 (± 0.2), 0.5 (± 0.09) and 0.3 (± 0.06) for macroplastics, microplastics, and cleanup, respectively (Tables S2 and S3). Our higher uncertainty in microplastic impacts is largely driven by a current lack of knowledge regarding impact intensity and its reversibility. The lower uncertainty for cleanup impacts is the result of extensive data collection by The Ocean Cleanup on interactions between marine life and their cleanup technology over the past years, as well as the direct expert experience from several of the authors.

**Fig. 4:**
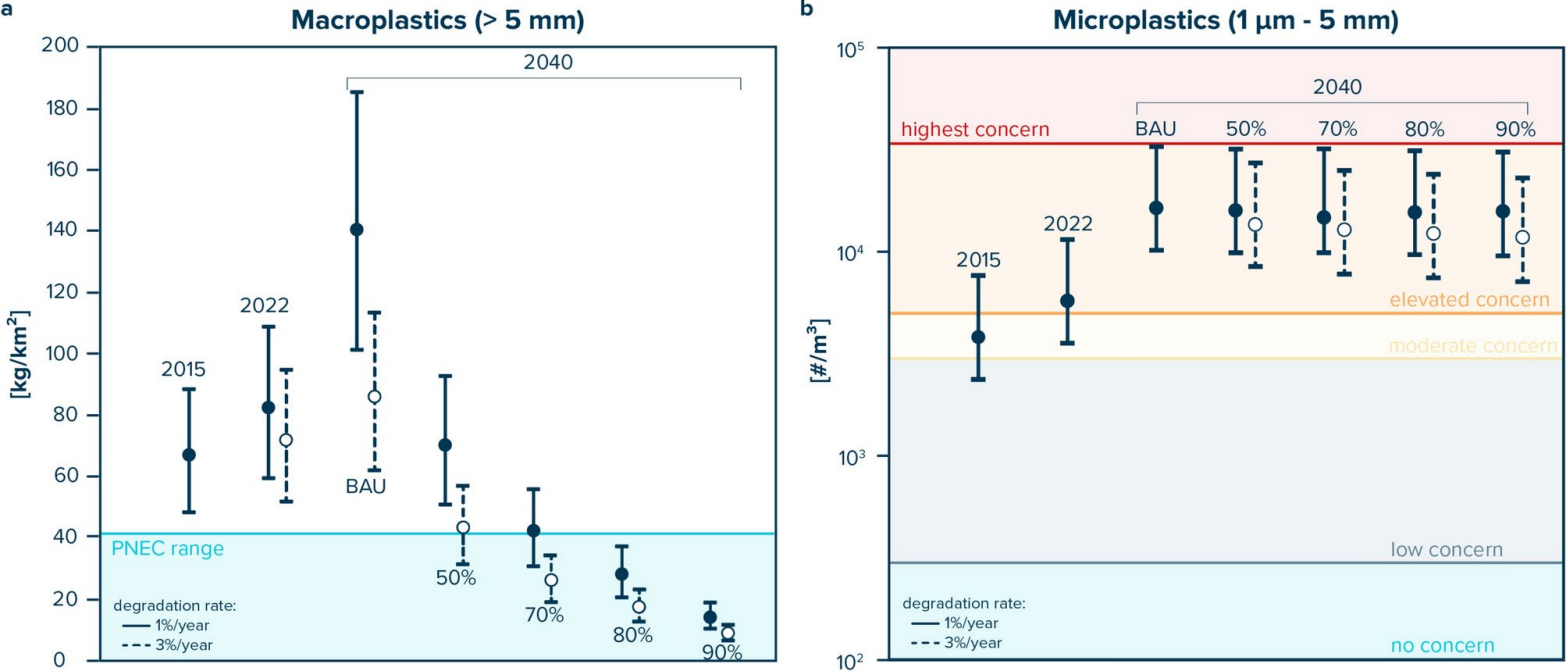
Predicted environmental concentrations (PEC) of (**a**) macroplastics (> 5 mm) and (**b**) microplastics (1µm–5 mm) in the North Pacific Garbage Patch based on low (1%/year; solid vertical lines) and high (3%/year, dashed vertical lines) macroplastic degradation rates. For 2040, results are presented for a business-as-usual scenario (BAU), as well as for scenarios with 50%, 70%, 80%, and 90% macroplastic cleanup. (**a**) Blue shaded area represents the range of predicted no effect concentrations (PNEC) for macroplastic entanglement (marine mammals, sea turtles, and seabirds)^24^. (**b**) Horizontal lines represent proposed microplastic toxicity thresholds for food dilution (particle size range: 1 µm–5 mm)^60^.

For the year 2022, our model predicts average macroplastics concentrations of 72 kg/km^2^ (52–95 kg/km^2^) under a high degradation scenario (4% annual growth, 3% annual degradation, resulting in a 1% net growth; see methods) and 83 kg/km^2^ (60–109 kg/km^2^) under a low degradation scenario (4% annual growth, 1% annual degradation, resulting in a 3% net growth; see methods). Additionally, the model estimates average concentrations of large microplastics (500 µm–5 mm) at 1,020,024 #/km^2^ (634,965 #/km^2^–2,045,585 #/km^2^) or 5,741 #/m^3^ (3,574 #/m^3^–11,514 #/m^3^) when rescaled to microplastics between 1 µm and 5 mm in size using probability density functions and converted to volumetric concentrations (Fig. 4). Note that these concentrations are likely conservative, as our model does not account for potential removal of microplastics from the ocean surface.

Estimated current levels of macroplastic pollution in the NPGP therefore exceed reported predicted no effect concentrations (PNECs) for seabirds, marine mammals, and sea turtles^24^. Under business-as-usual scenario (i.e., no cleanup), macroplastic concentrations could reach 86 kg/km^2^ (62–114 kg/km^2^) or 140 kg/km^2^ (101–185 kg/km^2^) in 2040 in our high and low degradation scenarios, respectively (Fig. 4). In a scenario with a 50% cleanup of macroplastics, predicted macroplastic concentrations in 2040 fall within the PNEC range^24^ for marine mammals and sea turtles in our high degradation scenario. With a 80% macroplastic cleanup, both the low and high degradation scenarios for 2040 fall within the reported PNEC range of macroplastics for these species (Fig. 4). Predicted macroplastic concentrations, however, remain above the reported PNEC values for seabirds (0.3–0.9 kg/km^2^)^24^ in all scenarios.

We further find that current average microplastic concentrations in the NPGP exceed the elevated concern threshold for microplastics toxicity of 5000 #/m^3^^60^ (Fig. 4). In 2040, the upper confidence interval for our predicted average microplastic concentrations approaches the highest concern microplastics toxicity threshold of 34,000 #/m^3^^60^. The predicted average microplastic concentration decreases across all our cleanup scenarios, with the greatest reduction observed in the high degradation scenario. However, in all cases, concentrations remain above the elevated concern threshold.

### Greenhouse gas emissions

Based on demonstrated performance of The Ocean Cleanup’s System 03, we estimate that a 10-year full-fleet cleanup emits approximately 290 kilotons of carbon per year of cleanup, corresponding to cumulative carbon emissions of 2.9 million metric tons of carbon (Mt C) (Table S10). Implementing achievable improvements to the current cleanup operations (more efficient vessels, fewer transits, increased operational uptime and a longer system of 2.5 km), however, results in a 67% reduction of carbon emissions, with an estimated 96 kilotons of carbon per year of cleanup and a total of 1.0 Mt C over the cleanup period of 10 years (Table S10). More efficient targeting of plastic hotspots within the NPGP (through the use of drones, satellites and drifters) is predicted to further reduce carbon emissions to 85 kilotons of carbon per year of cleanup and to shorten the cleanup period to 5 years, thus resulting in cumulative emissions of 0.4 Mt C over the duration of the cleanup (Table S10).

**Fig. 5:**
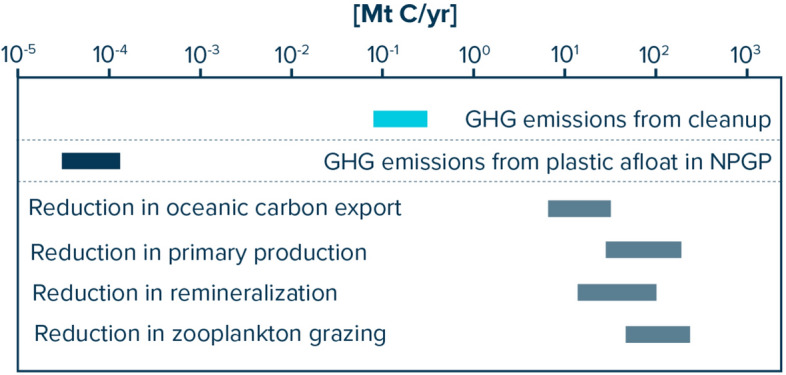
Estimated carbon cycle impacts in million metric tons of carbon (Mt C) per year in the North Pacific Garbage Patch (NPGP). Values for cleanup (light blue) represent greenhouse gas (GHG) emissions from vessels during cleanup (Table S10), while photodegradation (dark blue) represents GHG emissions from floating plastics in the NPGP due to solar UV radiation. Grey bars indicate estimated reduction in respective carbon fluxes in the NPGP due to microplastic pollution, with the ranges indicating impacts over 10 (low) and 100 (high) years, respectively (Table S11).

Concerning the plastic mass predicted to be afloat in the NPGP, we estimate that photodegradation of floating plastic debris currently emits approximately 32–122 t C in the form of GHGs per year (Fig. 5). Annual GHG emissions from floating plastics are therefore about three orders of magnitude lower than predicted carbon emissions from the cleanup vessels.

### Impacts on ocean carbon cycling

Applying the biogeochemical model from Richon et al.^28^ to evaluate the potential impacts of microplastics on zooplankton grazing rates and their consequences for key biogeochemical processes, we estimate that in the NPGP, microplastic pollution could result in a 27–55% decrease in primary production, a 30–77% decrease in organic matter remineralization and a 34–63% decrease in zooplankton grazing (Table S11). Overall, these changes are estimated to reduce carbon export in the first 100 m of the NPGP by 30–65% (Table S11). Such a reduction corresponds to a modeled carbon export decrease of 7–13 Mt C per year and 15–30 Mt C per year after 10 years and 100 years, respectively (Fig. 5). Potential microplastics impacts on ocean carbon export could thus be two orders of magnitude larger than carbon emissions during cleanup.

## Discussion

Our assessment based on currently available data and expert judgement suggests that wildlife in the North Pacific subtropical gyre generally shows higher vulnerabilities to macro- and microplastic pollution than to cleanup efforts. This lower vulnerability to cleanup is largely driven by the smaller geographical extent and shorter duration of the impacts. Cleanup activities target a specific sub-area within the NPGP, the so-called plastic hotspot territory (corresponding to ~ 50% of the NPGP surface area^61,62^), and they are intended to be limited in duration (~ 5–10 years). Impacts of plastic pollution afloat in the NPGP, on the other hand, have a wider geographical extent (extending to both coastal environments adjacent to the NPGP due to escape from the NPGP as well as to the deep sea underneath the NPGP due to fragmentation into microplastics and subsequent sinking) and longer duration (e.g., decades to centuries). Although the intensity, reversibility, and frequency of the cleanup activities could be similar to the pollution itself, our findings indicate that removing existing macroplastic pollution from the NPGP is beneficial to marine life living in or migrating through the area, particularly when considering long-term ecological impacts of plastic pollution. The transfer of small plastic fragments from the NPGP into the underlying deep sea^38^ further suggests that the benefits of cleaning the NPGP extend to the deep-sea ecosystem.

An 80% removal of macroplastics compared to business-as-usual is estimated to substantially decrease the risk of entanglement, resulting in macroplastic concentrations within predicted safe levels for marine mammals and sea turtles in both of the future scenarios. Notably, even under an 80% macroplastic cleanup scenario, modeled concentrations remain above established ecological risk thresholds for seabirds, underscoring their heightened vulnerability to macroplastic pollution and the need for species-specific conservation interventions. The exact break-even point, referring to the % reduction in macroplastic pollution at which the negative impacts of the cleanup start to exceed its benefits, should be evaluated in future research as more information becomes available.

Cleanup could also reduce the risk of microplastic concentrations exceeding the highest concerns management threshold in 2040 due to the mitigation of macroplastic fragmentation at sea. In the mid- to long-term, this reduces the risk for species with high vulnerability towards microplastics, such as zooplankton, fish, and elasmobranchs. We note, however, that microplastic concentration remains above the elevated concern threshold in all our scenarios. This is because the majority (74–96%) of the observed microplastic accumulation in the NPGP results from external inputs rather than from the fragmentation of floating macroplastics in the region^62^. The projected rise in microplastic concentrations in the NPGP is primarily driven by the fragmentation of macroplastics in coastal and land-based environments, followed by the offshore transport of the resulting microplastics^21,34,62–66^. Therefore, to effectively reduce microplastic accumulation in the NPGP and its associated risks to marine life, both the cleanup of floating macroplastics in the NPGP and the removal of legacy macroplastic pollution in coastal areas—such as beach cleanups and river interception—are essential.

Marine mammals, seabirds, and zooplankton show no substantial negative impacts by the cleanup operations. Particular attention during cleanup should be given to neuston, fish, sharks, and sea turtles, which show medium vulnerabilities towards cleanup. To date, no systematic impacts on neuston have been observed during cleanup operations^67^ (Fig. S1). Furthermore, neuston inside the NPGP often show similar or lower abundances within the hotspot territory compared to the wider NPGP^61^. Targeting cleanup on the hotspot territory could therefore minimize interactions with neuston during cleanup.

Although fish dominate the primary incidental catch during cleanup (~ 84% by count, Table S4), their bycatch consists of several coastal (often reef-associated) and thus potentially invasive species and species with an IUCN (International Union for Conservation of Nature) status of Least Concern (> 99%). This suggests that their capture during cleanup likely does not pose substantial impacts on fish populations naturally occurring in the NPGP as a whole. The relatively high vulnerability of elasmobranchs towards cleanup is mainly (~ 98%) due to interactions with pygmy and cookiecutter sharks (Dalatiidae), which are classified as Least Concern by the IUCN, and blue sharks (*Prionace glauca*; ~ 1%), which are classified as Near Threatened. Species classified as Vulnerable or Endangered, such as sea turtles (e.g., loggerhead, green and olive ridley), have also been encountered as incidental catch during cleanup. Cleanup efforts need to closely monitor impacts on these critical species and develop additional mitigation measures to ensure negative interactions are reduced. In addition to marine fauna observers, marine animal safety hatches and underwater cameras are promising tools^68^. Negative interactions occur when an animal becomes trapped in the plastic accumulation area of the cleanup system, specifically at the back of the retention zone. Installing a safety hatch that can be activated to close off this area prevents further animal entry upon detection via underwater cameras. Enhancing detection capabilities with artificial intelligence can further support the identification of species of concern, enabling timely intervention to minimize harm. Such a system has already been successfully tested during cleanup operations in the NPGP by The Ocean Cleanup.

So far, all examined sea turtles incidentally captured during cleanup in the NPGP were found to have ingested plastics, and some sea turtles were rescued from entanglement in plastic debris upon sighting during cleanup operations^69^, supporting earlier findings that turtles residing in the NPGP are at risk of suffering impacts from plastic pollution in these offshore waters^24,70^. Thus, if done cautiously, removing plastic afloat in the NPGP can have a substantial positive impact on sea turtles by both preventing entanglement and freeing some individuals found entangled in ghost gear.

Cleanup efforts in the NPGP could have a negative impact on octopuses and rafting species. These animals benefit from macroplastics as shelter and habitat and, due to their direct association with plastic debris, exhibit a high vulnerability towards cleanup. However, a majority (~ 80%) of rafting species present on plastics in the NPGP have a coastal origin^31^. Open ocean species are thus increasingly in direct competition with, or even preyed upon, by non-native and potentially invasive species that are transported to the NPGP on plastic items originating in coastal areas. The long-term ecological and environmental impacts of the presence of rafting coastal communities on marine life in the NPGP and whether removing them could benefit native species remains unknown. It is clear, however, that cleaning up the NPGP decreases the amount of harmful plastic debris and associated non-native rafting species ‘spilling’ from the NPGP to other areas such as the Hawaiian Archipelago, including the Papahānaumokuākea Marine National Monument, one of the world’s largest marine protected areas^71,72^.

There is evidence that smaller and partially degraded plastic fragments with higher surface-to-volume ratios undergo further degradation proportionally faster, as photodegradation affects the plastic surface^22,73^. It is, therefore, likely that overall GHG emissions will increase over time due to weathering and fragmentation of macroplastics present in the NPGP. Conversely, smaller plastic particles can hetero-aggregate with natural particles and organic matter, ultimately removing them from the ocean surface and consequently the potential for photodegradation is minimized. Biofouling can further shield some of the plastic particles’ surface from UV radiation. Taken together, our estimates of photocatalytic GHG emissions from plastics afloat in the NPGP need refinement in the future. Nevertheless, our results indicate that GHG emissions avoided by removing plastics from the NPGP are unlikely to exceed GHG emissions occurring during cleanup when using cleanup vessels powered by fossil fuels.

Nevertheless, our analysis reveals that cleanups could reduce microplastics impacts on the biological carbon pump by preventing the fragmentation of macroplastics into smaller particles. We show that in an oligotrophic plastic accumulation zone like the NPGP, microplastics could substantially impact carbon export fluxes, including through the disruption of zooplankton grazing. From an ecological perspective, this is concerning because such oligotrophic ecosystems tend to have exceptionally tight linkages between trophic levels with a strong dependence on nutrient recycling sustaining proportionally large quantities of predators. From an ocean carbon sequestration perspective, the potential magnitude of carbon flux perturbation reported here is concerning if microplastic contamination proves persistent over multi-century timescales (the operational timescale of the biological carbon pump on climate)^74–76^. We note, however, that zooplankton grazing parameterizations remain a major source of uncertainty in biogeochemical models^77^. Second, ecological shifts due to changes in particle sinking rates^78^ may also impact community calcification rates and hence the ocean carbon buffer and air-sea CO_2_ exchange^79^. The overall impact of microplastic pollution, including further breakdown to nanoplastics and leaching of additives and chemicals, on carbon uptake and cycling in the NPGP therefore remains unclear. We advocate for more quantitative assessments of the impacts of plastic pollution, particularly microplastics, on primary production, zooplankton grazing, and associated biogeochemical processes such as air–sea CO_2_ exchange and carbon export. Such research should incorporate key microplastic characteristics, including size, shape, polymer composition, and density, which are known to influence their environmental behavior and interactions. In addition, the potential for microplastics to alter air–sea CO_2_ exchange via stimulation of biological surfactant production^80,81^ and subsequent modification of the sea surface microlayer warrants further investigations.

In summary, our assessment suggests that if done with appropriate safeguards, removing legacy macroplastic pollution accumulated in the NPGP provides immediate benefits to marine life, reduces adverse future impacts of secondary microplastics on marine life and oceanic carbon cycling, and decreases the plastic toxicity debt. Furthermore, cleaning the NPGP may also positively contribute to increasing awareness and reducing beach cleanup costs and potential tourism revenue loss on the Hawaiian Islands impacted by plastics ‘spilling’ from the NPGP. Lastly, cleanup operations in the NPGP also represent a unique opportunity to conduct scientific research in a remote and difficult to access region (Table S12). However, cleaning the NPGP could come at a climate-cost, with an estimated 0.4–2.9 million metric tons of carbon. The extent to which a cleaner and thus healthier North Pacific gyre ecosystem impacts the future uptake of anthropogenic CO_2_ emissions is not fully understood. Our results suggest that removing macroplastics and can help reduce future levels of microplastic accumulation in the NPGP, thereby reducing impacts on ocean carbon export—potentially outweighing cleanup-related carbon emissions by orders of magnitude. However, considerable uncertainties remain, particularly regarding how predicted changes in carbon export influence air-sea CO₂ exchange. Ultimately, cleanup-related carbon emissions should be evaluated based on their net impact on atmospheric CO₂ rather than focusing solely on changes within the ocean carbon cycle. Due to limited and uncertain data, the overall climate impact of cleaning the NPGP remains unknown. However, this initial assessment provides the foundation for a framework that can be iteratively refined as further research reduces uncertainties in the estimates.

We emphasize that parallel measures aimed at managing plastic production and reducing plastic emissions into the environment are needed to make cleanup operations unnecessary in the longer term (i.e., to avoid cleaning in perpetuity). For the NPGP, this includes reducing plastic emissions from land-based sources^82^ and industrial fishing activities^20^, as well as reducing generation of secondary microplastics in coastal environments and their subsequent transport offshore through cleanup of beached macroplastics and interception in rivers. Here, data collected during cleanup on the quantity, composition, and origin of plastics helps to formulate and inform strategies. It also provides data-based insights into the success or failure of midstream and upstream policies, including their accountability and effectiveness. Cleanup is a beneficial downstream strategy that complements the upstream efforts to reduce the production and use of plastics.

With this work, we set a precedent for how environmentally sound removal of existing plastic pollution, as referred to in the current draft of the global Treaty to End Plastic Pollution^50,51^, can be defined in practice. Our NEBA framework can further be used to assess the net environmental impacts of different cleanup technologies and to measure improvements toward their optimal environmental approaches, such as improved targeting of plastic pollution hotspots, electrification of cleanup vessels, continued data collection from cleanup sites, and detection and avoidance of marine species. It also underscores the complexity of assessing cleanup impacts. Cleanup organizations often lack the data and capacity to conduct comprehensive assessments at this scale. To establish science-based, universal industry standards for sustainable cleanup operations, cross-sectoral collaboration between policymakers, researchers, non-profits, and industry stakeholders is essential. Such standards can contribute to a common understanding of the possible negative effects caused by cleanup efforts, the relevance of which can further be evaluated by considering local, traditional, and Indigenous Knowledge, and by engaging scientists, the civil society, and the private sector, as recommended in the current draft for a Global Plastics Treaty.

## Conclusions

Our analysis shows that removing legacy macroplastic pollution already accumulated in the NPGP benefits marine life in the area, even when taking into account the factors of GHG emissions and bycatch. Cleanup may also reduce the possible long-term impacts of (macro and micro) plastic pollution on regional carbon cycling, yet the direct comparison of cleanup emissions to oceanic carbon flux changes remains uncertain. The framework presented here allows for continuous evaluation and revision of resulting outcomes as new information becomes available. We therefore recommend the periodic reassessment of our initial findings and advocate that similar net environmental impact assessments are conducted for cleanup approaches in other environments. The NPGP is located in the high seas, meaning it falls outside of the economic zones regulated under national jurisdictions that require environmental impact analyses to be conducted before the implementation of any major project interfering with marine ecosystems. The impact assessment framework developed here can contribute to creating industry standards on operational efficiency and impacts on marine ecosystems that can be included in relevant policy, including the High Seas and Global Plastics Treaties.

## Methods

### Cleanup approach

The assessment of cleanup impacts is based on the approach and technology developed by The Ocean Cleanup. Here, we focused on their latest ocean technology, i.e., System 03^68^. The system consists of a retention zone attached to a floating barrier and is towed between two slow-moving (< 2.5 knots) vessels (Fig. 2). The barrier is approximately 2.2 km long and suspends a screen with a 16 mm mesh extending from the surface to 4 m below the surface of the water, where most floating plastic is encountered^38,83,84^. The retention zone is 70 m long, with a 15 m wide opening that funnels to a 5.5 m wide plastic retention section, composed of 10 mm × 10 mm square mesh in the inner netting, and a 50 mm × 50 mm square mesh in the outer netting. Microplastics are thus not targeted by the cleanup device. Using monitoring data, numerical modelling of plastic dispersal, and artificial intelligence, the system is steered towards areas within the NPGP identified as having elevated densities of floating plastic debris (i.e., “hotspot territory”^61,62^). Floating plastic debris is guided by the wings towards the retention zone. Once the retention zone is ready to be emptied (on average after ~ 4 days), it is pulled onto the vessel and the plastic is emptied on deck for sorting and packing. Subsequently, the retention zone is returned to the water, and the process begins again. Back onshore, the majority (> 97%) of the plastic catch enters the recycling stream into durable products, with the remaining < 3% discarded as waste.

The Ocean Cleanup has commissioned an independent Environmental Impact Assessment (EIA) for each iteration of their offshore cleanup technology^67,85,86^. Each EIA identified potential negative impacts of the technology on the environment and defined a series of mitigation measures to minimize identified risks. These preventive actions included measures built into the design of the technology (design measures), measures based on how the system is operated (operational measures), as well as measures based on monitoring (monitoring measures) (Fig. 2)^69^, all guided by a comprehensive Environmental Management Plan (EMP). As part of their EMP, The Ocean Cleanup collects data on incidental catch of marine life and monitors interactions between wildlife and the cleanup technology. Here, we use their data collected throughout 18 cleanup campaigns between July 2021 and November 2023 to evaluate cleanup impacts on marine life. All data are available in the Supplementary Information (Tables S1–S4).

### Marine life vulnerability assessment

To assess impacts of macroplastics, microplastics, and cleanup actions on marine life, including both direct impacts such as entanglement and ingestion as well as indirect impacts such as plastic-associated chemicals and organisms, we used previously developed methodologies^87–89^ to evaluate the sensitivity of several key ecological guilds: zooplankton, obligate neuston, bony fish, elasmobranchs, sea turtles, seabirds, marine mammals, cephalopods, and rafting species (Fig. 1). Note that neuston (a collective of species living at the surface of the ocean, often referred to as floating life) and rafting species (species that live on or are attached to plastics) were included as two distinct categories given their potential relevance related to impacts associated with cleanup operations^31,47,49,61,90,91^. Subsequently, we evaluated the vulnerability of each ecological guild to macroplastics, microplastics and cleanup in the NPGP (broadly defined as the region between 160°W–125°W and 20°N–45°N to allow for geographical variability of the NPGP^19,20^) by individually scoring the extent (i.e., geographical scale), duration (i.e., the persistence of the stressor), intensity (i.e., health effects), reversibility (i.e., capability of an organism to recover from the stressor), and frequency (i.e., abundance of interactions between stressor and individuals) of the impact (Table S9)^80,81,84^.We further included a measure of certainty that allowed these scores to be qualified by the level of certainty in the existing knowledge^88^.

All impact measures (i.e., extent, duration, intensity, reversibility, frequency) were scored on a scale of 1 to 3, with 1 representing the lowest possible level of impact and 3 being the highest level (Table S9). Note that we chose this narrow scale because detailed knowledge on plastic pollution impacts often remains limited. As more data becomes available, the scale can be extended in future assessments, thus providing more detailed insights. Associated certainties were scored on a scale of 1 (low confidence) to 5 (high confidence) (Table S9).

The scoring was based on our best available assessment of current knowledge derived from published literature (see Supplementary Information for a detailed list of references considered in this study), data collected by The Ocean Cleanup (Tables S4–S8), and expert judgement of the authors. First, a score for each impact measure was proposed by the first author and accompanied by a detailed description of the reasoning and relevant literature and data (see Supplementary Information). To minimize subjectivity, the scoring assessment was then reviewed by all authors, and revised in multiple rounds of revisions until consensus among all authors was reached. It is important to note that the scoring presented here reflects our best judgement given the current uncertainties and data limitations. We further note that the scoring could be influenced by the specific expertise represented by the authors. We therefore encourage the periodic reassessment of our initial findings by the scientific community to iteratively decrease uncertainty as new data become available.

Final vulnerability scores for each ecological guild were derived by considering the total impact an ecological guild is expected to experience. An expected value, in the statistical sense, is the product of the probability of an impact and its severity. There is insufficient data to determine a complete probability distribution for impacts at different levels. Therefore, we approximated this quantity using the simple expert scoring described above for the individual impact metrics. The severity of the impact is captured by the intensity and reversibility. The probability of the impact should be proportional to the areal extent of the stressor, to its temporal extent (duration), and to the chance of the stressor encountering an organism (frequency). This suggests an expected vulnerability score as the product of the five main impact metrics. Since the quantities are unit-less, we took the 5th root (i.e., the geometric mean), to keep the range more manageable (Eq. 1):

1 $$Vulnerability= \prod_{i=1}^{n}{{(impact}_{i})}^\frac{1}{n}$$

where ${impact}_{i}$ represents the score of the respective impact measure (n = 5; i.e., extent, duration, intensity, reversibility, frequency). The resultant vulnerability score can vary between 1 and 3. In line with previous environmental impact assessments^88,89,92^, we chose equal weighting for impact measures, as a high score in any single impact measure could potentially result in ecological harm. However, weighting could easily be adjusted in future assessments if a given impact measure becomes known to play a larger role in determining marine life vulnerability.

Each expected vulnerability score is accompanied by an uncertainty score. The combined uncertainty score is obtained similarly as the geometric mean of the reciprocals of the certainty scores for each impact measure (Eq. 2):

2 $$Uncertainty= \prod_{i=1}^{n}{(\frac{1}{{certainty}_{i}})}^\frac{1}{n}$$

where ${certainty}_{i}$ represents the score of the associated certainty score for each respective impact measure (n = 5; i.e., extent, duration, intensity, reversibility, frequency). This uncertainty score is analogous to the standard deviation of the product of independent random variables with zero means being the product of the individual standard deviations. Note that we took the reciprocals of the certainty scores, since a high certainty score is indicative of low uncertainty. Uncertainty, thus, can range from 0.2 (high confidence) to 1.00 (low confidence).

This scoring approach allowed for vulnerabilities to be ranked and compared based on expert assessment of ecological impacts, consequently providing information on the relative sensitivity of each ecological guild to the respective anthropogenic stressor (e.g., macroplastics, microplastics, and cleanup). However, it is important to note that the approach implemented here cannot be used to quantify differences in vulnerability in absolute terms^87^. In other words, vulnerability scores of 2 and 1 for ecological guilds X and Y, respectively, indicate that guild X is more vulnerable to the respective anthropogenic stressor than guild Y, but it does not mean that guild X is two times more vulnerable than guild Y.

### Ecological risk assessment

To complement our vulnerability framework with a risk assessment framework for macro- and microplastic impacts, we compared current and future predicted environmental concentrations (PECs) of plastics in the NPGP to predicted no-effect concentrations (PNECs). The PEC values for macro- and microplastics were estimated for the years 2015, 2022 and 2040. As a baseline for 2015, we used reported average NPGP macro- and microplastics concentrations by Lebreton et al.^19^ of 67 kg/km^2^ (lower–upper estimate: 48–89 kg/km^2^) and 678,000 #/km^2^ (422,000–1,360,000 #/km^2^). Note that the microplastic concentrations reported by Lebreton et al.^19^ were derived by deploying a Manta trawl with a net mesh size of 500 µm. They subsequently divided the number of particles (500 µm–5 mm) found in the Manta trawl sample by the trawled area (trawl width multiplied by the trawled distance). To allow for comparison with reported microplastics toxicity thresholds^60,93^, we converted their areal microplastic concentrations (i.e., #/km^2^) into volumetric concentrations (i.e., #/m^3^) by dividing them by the Manta trawl height (0.15 m). Next, we rescaled these concentrations to microplastics between 1 µm and 5 mm in size using probability density functions^94,95^ and a corresponding power law exponent of 2.07^96^.

Macroplastic concentrations in 2022 and 2040 were predicted assuming a net annual mass growth rate for macroplastics between 1% (high degradation scenario) and 3% (low degradation scenario) (Table S10). These net growth rates were based on an annual global growth rate of 4%^9^, and assuming that between 1% and 3% of macroplastics fragment into smaller particles per year^14,22,97^. We then estimated macroplastic concentrations in 2040 in a business-as-usual scenario (i.e., no cleanup), as well as for different cleanup scenarios^19^ (i.e., a 50%, 70%, 80% and 90% reduction of the macroplastic mass predicted to be afloat in the NPGP by 2040 without the remediation) (Table S9).

For future predictions of microplastic concentrations, we used the NPGP plastic mass balance model developed by Lebreton et al.^62^. These authors observed a net growth rate for numerical concentrations of microplastics in the NPGP hotspot territory of 6% per year between 2015 and 2022^62^. They further found that the observed increase in microplastic concentrations in the NPGP cannot only result from the degradation of macroplastics that were already present in the NPGP but instead likely requires additional inputs of external microplastics to the region. Based on annual degradation rates of macroplastic of between 1 and 3%, their mass balance model suggests that between 74 and 96% of the observed increase in small plastic fragments (0.5–50 mm) could result from external inputs rather than from the fragmentation of macroplastics present in the NPGP. We therefore run two scenarios assuming either 74% or 96% external inputs of microplastics to the NPGP. We subsequently reduced microplastic inputs generated by macroplastics by 50%, 70%, 80%, and 90% to estimate microplastic concentrations in 2040 (Table S9). Cleanup, however, did not impact the estimated inputs of external microplastics, as these microplastic inputs are not mitigated by removing macroplastics afloat in the NPGP.

Macroplastic entanglement hazardous threshold effect concentrations (HC_5_; the macroplastic concentration at which 5% of the population is annually entangled) were previously reported to range between 1.7–4.7 kg/km^2^, 0.05–207.0 kg/km^2^, and 10.3–189 kg/km^2^ for seabirds, marine mammals, and sea turtles, respectively^24^. After applying an assessment factor of 5 as recommended by EU legislation^98,99^, this corresponds to PNEC values for macroplastics of 0.3–0.9 kg/km^2^ for seabirds, 0.01–41.4 kg/km^2^ for marine mammals, and of 2.1–37.8 kg/km^2^ for sea turtles. Note that such PNEC values only consider entanglement risk but no impacts from macroplastic ingestion, and are therefore likely to underestimate the total risk of macroplastics.

For microplastics, we used four microplastic management thresholds as derived from a recent meta-analysis in which a broad array of toxicological studies were incorporated into a risk management framework^60^. Specifically, we applied the microplastic toxicity thresholds for food dilution (particle size range: 1 µm–5 mm), which were defined as 300 #/m^3^ (threshold 1: low concern; management recommendation: increase monitoring frequency), 3000 #/m^3^ (threshold 2: moderate concern; investigate sources of contamination), 5000 #/m^3^ (threshold 3: elevated concern; initiate mitigation strategies), and 34,000 #/m^3^ (threshold 4: highest concern, implement pollution control measures), respectively^60^. We note that these thresholds are associated with large uncertainties due to the limited quality of microplastics effects data at present and should thus be re-evaluated as new data become available.

### Carbon impact assessment framework

The cleanup technology developed by The Ocean Cleanup requires the use of motorized vessels, which results in carbon emissions into the atmosphere. Here, we assume three scenarios to estimate carbon emissions resulting from the cleanup (Table S10). The first scenario is based on current System 03 performance and operations. The second scenario assumes simple-to-implement improvements to System 03 operations such as using vessels with lower fuel consumption, longer vessel rotations of 12 weeks instead of 7 weeks (resulting in fewer transits from and to the NPGP), more than two vessels working in rotation, a 10% increase in operational uptime, larger extractions of 40 tons and an increase of the system’s barrier from 2.2 to 2.5 km. The third scenario further assumes more efficient targeting of plastic hotspots within the NPGP, thus increasing the efficiency of the cleanup. Each scenario was run assuming an annual mass growth rate for macroplastics of 4% and macroplastic degradation rates of 3% and 1%, resulting in a net macroplastic growth rate of between 1% (high degradation scenario) and 3% (low degradation scenario) per year. Based on these scenarios, we estimated that removing around 80% of the total macroplastic mass in the NPGP can be achieved by operating ~ 54 cleanup systems for 10 years (scenario 1), 20 cleanup systems for 10 years (scenario 2), or 18 systems for 5 years (scenario 3) (Fig. S2, Table S10). We subsequently estimated annual carbon emissions per year of cleanup as well as cumulative carbon emissions based on estimated fuel consumption and by assuming that the burning of one ton of fuel results in 3.2 tons of CO_2_ emissions. To assess the net environmental impact of these carbon emissions, we compared them with plastic pollution impacts on carbon cycling in the NPGP, including (i) impacts on GHG emissions related to the weathering of plastics afloat in the NPGP, and ii) the interference of microplastics with ocean carbon uptake and export.

### Greenhouse gas emissions from photodegradation

Plastic at the sea surface is exposed to solar UV, which causes weakening of the polymer matrix and photooxidation, thus accelerating fragmentation of floating plastic debris^22,100,101^. Photodegradation primarily leads to the generation of smaller daughter products, ranging from small molecules to mono- and oligomers as well as nanoplastics that remain in seawater^22,102^. Additionally, photodegradation also leads to the formation of GHGs^22,73^. Laboratory-based experiments on polyethylene and polypropylene (the two most common polymers found afloat in the NPGP^19,66,103^) using virgin polymers and plastics collected from the NPGP showed that between 1.7 and 2.3% of their mass is degraded annually by solar UV radiation as encountered at the surface of the subtropical and tropical ocean^22^. Greenhouse gases have been estimated to account for ≤ 5% of this mass loss^22,73^.

Here, we estimated GHG emissions from plastics afloat in the NPGP based on the estimated plastic mass loading in 2015^19^, and assuming photodegradation rates by Delre and co-workers^22^. The lower estimate was derived using the low estimate for predicted plastic mass loading (i.e., 45,000 tons) and a photodegradation rate of 1.7% mass loss per year, while the upper estimate was based on the high plastic loading (i.e., 129,000 tons) and a photodegradation rate of 2.3% mass loss per year. The total annual mass loss of plastics in the NPGP due to photodegradation was thus subsequently converted to carbon emissions, assuming that GHGs contribute to 5% of the UV-degraded plastic mass as demonstrated for plastics collected from the NPGP^22^ and an average carbon content for polyethylene and polypropylene of 82.4%^104^.

### Microplastic impacts on ocean carbon uptake and export

The pelagic food web plays an important role in the biological pump, with carbon transported from the ocean surface to the deep ocean through the sinking of particles such as zooplankton, fecal pellets, sinking carcasses, marine snow, and phytoplankton detritus^105,106^. Microplastics have been shown to interact with key processes of the biological carbon pump, both in laboratory experiments as well as in in-situ measurements^37,78,107–113^. These observations indicate potentially large-scale impacts of microplastics on the biogeochemical cycling of carbon in the ocean^10,28^. First attempts to assess such potential impacts have been made using global biogeochemical models^10,26,28,75,79^.

Richon et al. explored the potential impacts of microplastics on zooplankton grazing rates and their consequences for the global ocean surface biogeochemistry based on various water contamination impact thresholds (i.e., microplastics to prey ratios varying between 0.1 and 0.9) and constant physical, biogeochemical and riverine plastic emissions forcings, using the NEMO/PISCES-PLASTIC coupled physical-biogeochemical model^26,114^. Here, we used the outputs from their global simulations and subsequently extracted their results for the NPGP (using the 1 kg/km^2^ microplastic threshold as defined by Lebreton and co-workers^19^). We then calculated the integrated microplastic impacts on zooplankton grazing, primary production, remineralization, and carbon export in the first 100 m of the NPGP on short (10 years) and long (100 years) timescales.

## Supplementary Information

Supplementary Information 1.

Supplementary Information 2.

## Data Availability

All data generated or analysed during this study are included in this published article and its supplementary information files.
