# Supplementary material for: Evaluating the environmental impact of cleaning the North Pacific Garbage Patch
Source: Sci Rep. 2025 May 27;15:16736. doi: 10.1038/s41598-025-00619-w (PMC12116914; doi:10.1038/s41598-025-00619-w)
Supplement: Supplementary file 2 — Supplementary Information 2. [file 41598_2025_619_MOESM2_ESM.pdf]

SUPPLEMENTARY INFORMATION

Evaluating the environmental impact of cleaning the North Pacific Garbage Patch

Matthias Egger[1,2*], Andy M. Booth[3], Thijs Bosker[4], Gert Everaert[5], Samantha L. Garrard[6], Vilma Havas[7,8], Helga S. Huntley[9], Albert A. Koelmans[10], Karin Kvale[11,12], Laurent Lebreton[1,13], Helge Niemann[14,15], Qiaotong Pang[16], Maira Proietti[1,17], Peter Puskic[1,18,19], Camille Richon[20], Sarah-Jeanne Royer[1], Matthew S. Savoca[21,22], Arjen Tjallema[1], Marjolein van Vulpen[1], Yanxu Zhang[16,23,24], Ziman Zhang[16], Denise M. Mitrano[25]

[1]The Ocean Cleanup, Rotterdam, The Netherlands
[2]Empaqtify, St. Gallen, Switzerland
[3]SINTEF Ocean, Trondheim, Norway
[4]Leiden University, Leiden, The Netherlands
[5]Flanders Marine Institute, Ostend, Belgium
[6]Plymouth Marine Laboratory, Plymouth, UK
[7]Salt Lofoten, Norway
[8]Aalborg University, Denmark
[9]Rowan University, Glassboro, NJ, USA
[10]Aquatic Ecology and Water Quality Management Group, Wageningen University, Wageningen, The Netherlands
[11]GNS Science, Avalon, Lower Hutt, New Zealand
[12]Aotearoa Blue Ocean Research, Lower Hutt, New Zealand
[13]The Modelling House Limited, Raglan, New Zealand
[14]NIOZ Royal Netherlands Institute for Sea Research, 't Horntje (Texel), The Netherlands
[15]Department of Earth Sciences, Faculty of Geosciences, Utrecht University, the Netherlands
[16]Joint International Research Laboratory of Atmospheric and Earth System Sciences, School of Atmospheric Sciences, Nanjing University, Nanjing, China
[17]Universidade Federal do Rio Grande (FURG), Rio Grande, Brazil
[18]Institute for Marine and Antarctic Studies, University of Tasmania, Hobart, Australia
[19]Center for Marine Socioecology, Hobart, Australia
[20]University Brest, CNRS, Ifremer, IRD, Laboratoire des Sciences de l'Environnement Marin (LEMAR), IUEM, Brest, France
[21]Hopkins Marine Station, Stanford University, Pacific Grove, CA, USA
[22]California Marine Sanctuary Foundation, Monterey, CA, USA
[23]Frontiers Science Center for Critical Earth Material Cycling, Nanjing University, Nanjing, China
[24]Earth and Environmental Sciences, Tulane University, New Orleans, LA, USA
[25]Swiss Federal Institute of Technology (ETH) Zurich, Zurich, Switzerland

*** Correspondence:** Corresponding Author matthias.egger@theoceancleanup.com

Impact scoring

This section contains a detailed description of the scoring of the extent, duration, intensity, reversibility and frequency of macroplastics (> 5 cm), microplastics (< 5 mm), and plastic cleanup impacts (taking The Ocean Cleanup as a case study) on nine ecological guilds in the North Pacific Garbage Patch (NPGP), and the current level of certainty associated with this scoring (Table S1). An overview and analysis of the scoring is provided in Tables S2 and S3. The scores were derived based on finding consensus between the authors, supported by literature and additional data collected by The Ocean Cleanup on incidental catch of marine life and monitored interactions between wildlife and the cleanup system (Tables S4-S8).

Extent and duration of plastic pollution impacts

The NPGP is rapidly accumulating floating plastic debris [1–3]. Some plastics, however, escape from the NPGP and beach onto the shores of the Hawaiian Archipelago and the west coast of North America [4–7]. Microplastics in the NPGP have further been shown to be transported away from the ocean surface into the underlying deep sea [8,9]. If not removed, plastic debris afloat in the NPGP can traverse through it and therefore impact ecosystems beyond the NPGP, with macroplastic impacts extending to coastal environments adjacent to the NPGP (value = 2, certainty = 4) and microplastic impacts extending to both coastal environments adjacent to the NPGP as well as to the deep sea underneath the NPGP (value = 3, certainty = 4).

Macroplastic debris afloat in the NPGP has been shown to persist for decades, slowly degrading and fragmenting into microplastics [10–12]. Concentrations of the latter have more than doubled between 2015 and 2022 [2,3], suggesting that degradation and transport mechanisms away from the surface waters [8,9,13–16] are slower than the combined input and in situ formation rates of microplastics. The fragmentation of legacy macroplastic pollution into microplastics is therefore expected to increase microplastic

pollution in the NPGP for decades to come [1,17]. Consequently, the impacts of macro- and microplastics are rated as long-term (value = 3, certainty = 3).

Extent and duration of cleanup

While plastic pollution is ubiquitous across the North Pacific subtropical gyre [18–21], it is not uniformly distributed at the sea surface. Instead, floating plastic occurs at higher concentrations in the NPGP compared to the wider North Pacific subtropical gyre. Within the NPGP, plastic concentrations show a high variability on a sub-mesoscale, forming so-called plastic hotspots with elevated concentrations [1–3,13,22–24]. To increase efficiency, cleanup operations by The Ocean Cleanup target the plastic hotspot territory, an area with elevated plastic concentrations which typically corresponds to approximately 50% of the total NPGP surface area [2,3]. The extent of cleanup impacts is therefore mainly limited to the NPGP hotspot territory (value = 1; certainty = 3). Based on current cleanup system performance and envisioned improvements, we predict that an 80% reduction of the plastic mass estimated to be present in the surface waters of the NPGP without cleanup will take approximately 5 – 10 years (Table S10), thus resulting in a mid-term duration for impacts associated with direct interactions of marine life and the cleanup systems (value = 2, certainty = 3).

Impacts on zooplankton

Macroplastics

At present, the impacts of macroplastic on zooplankton remain largely unknown. While entanglement and ingestion are unlikely to occur, it could be possible that the biofouling community (particularly filter-feeders) present on floating plastic debris in the NPGP [25] negatively affects zooplankton by eating it or by competition for food supply naturally limited in the oligotrophic subtropical gyre. The low observed macroplastics to zooplankton ratio in the NPGP of < 0.01 [3,26] indicates that impacts are likely rare (value = 1, certainty = 3). High reproductive rates of zooplankton further suggest that impacts are

likely reversible (value = 1, certainty = 1). In the absence of evidence on impact intensity, we conservatively assume a moderate intensity (value = 2, certainty = 1).

Microplastics

Laboratory-based studies reveal that interactions with microplastics predominantly have a negative impact on zooplankton, including decreased swimming and feeding capacity, reproduction, survival, and acute toxicity from potential leaching of additives and adsorbed pollutants [27–32]. Current levels of microplastic (1 µm – 5 mm) pollution in the surface waters of the NPGP (> 84,000 particles/m^3) [2,3] reach estimated median hazardous microplastic threshold effect concentrations (HC_5, ~75,600 particles/m^3) for aquatic species [33]. Thus, based on the reported HC_5 and measured microplastic concentrations in the NPGP, we assume a moderate impact intensity of microplastics on zooplankton in the NPGP (value = 2, certainty = 3). However, we note that laboratory-based studies typically use extremely high concentrations of microplastics and are done with exposure to (mostly) pristine polystyrene particles, while the microplastics present in the NPGP are dominated by polyethylene and polypropylene [1,13,21], often highly weathered [34] and containing high levels of additives and adsorbed pollutants [35–38]. Thus, translating laboratory results to adverse effects in the natural environment remains challenging due to a lack of in-situ confirmation of impacts [39,40].

Concentrations measured on plastics collected from the NPGP often have plastic/lipid fugacity ratios >1 for many pollutants when compared to concentrations measured in fish and seabirds from the region [38]. Such positive fugacity ratios indicate the possible transfer of plastic-associated chemicals (additives and adsorbed pollutants) to biota in the NPGP. Although it remains uncertain whether such a transfer occurs for zooplankton, it is possible that some accumulation of potentially toxic chemicals due to ingestion of microplastics could result in impacts in zooplankton that are at least difficult to reverse (value = 2, certainty = 1).

Microplastic interactions have been demonstrated in >90% of studied zooplankton taxa in laboratory-based studies [27,30,41]. The oligotrophic NPGP constitutes a particularly vulnerable region for microplastic impacts on zooplankton because of the high microplastic concentrations associated with very low plankton biomass [42,43]. Thus, this region presents a high probability for zooplankton to interact with microplastics. For example, ingestion of microplastics has been demonstrated in wild populations of zooplankton (including salps) in the Northeast Pacific Ocean, with frequency of occurrence ranging from 3% to 100% [44–46]. Therefore, interactions between zooplankton and microplastics in the NPGP are considered potentially frequent (value = 3, certainty = 3).

Cleanup

Dominant zooplankton species in the NPGP are typically smaller than the mesh of the cleanup system's wings (i.e., < 16 mm) and retention zone (i.e., < 5 mm) and should thus be able to bypass the cleanup system with no harm [47]. However, some zooplankton can be temporarily displaced in the water column due to the wake created by the cleanup system. Cleanup impact intensity for zooplankton is therefore rated as low (value = 1, certainty = 3). While entrapment in the cleanup system is difficult to reverse, wake-associated vertical displacement in the water column likely is reversible. Given that entrapment is unlikely due to the large mesh of the system, the overall impact reversibility is likely dominated by vertical displacement and thus rated as reversible (value = 1, certainty = 2). Given the ubiquitous presence of zooplankton in the NPGP and their inability to swim away from an approaching cleanup system, interactions between zooplankton and the cleanup systems are rated as frequent (value = 3, certainty = 1).

Impacts on neuston

Macroplastics

The implications of encounters with floating macroplastics on neuston remain uncertain, but some physical injuries upon contact with rigid macroplastics could be possible due to their fragile bodies.

Neuston could also be impacted by plastic-rafting species either through direct predation or by increased competition for food, as well as by pathogens present on macroplastics [5,25,48–50]. Some evidence for direct predation of *Janthina janthina* by rafting anemones (L. Haram, personal communication) has been observed. However, in the absence of more data on direct predation, we conservatively assume a moderate impact intensity (value = 2, certainty = 1).. Observed macroplastics to neuston ratios in the NPGP are typically around one [1,3,26], indicating that encounters between macroplastic rafting communities and neuston are likely rare (value = 1, certainty = 3). Some neuston taxa have been observed to beach onto coastlines in high numbers [51], indicating possible high reproduction patterns. We therefore assume that impacts on neuston populations are reversible (value = 1, certainty = 1) but note that knowledge on reproduction rates of neuston currently remains scarce.

Microplastics

The extent of microplastic ingestion by neuston and the associated implications are currently unknown. It is possible that ingestion of microplastics and associated chemicals could have sublethal effects for neuston, similar to those demonstrated for certain zooplankton species [28,29]. We therefore assume a low impact intensity (value = 1, certainty = 1) and that some impacts could at least be difficult to reverse (value = 2, certainty = 1). Microplastic-to-neuston ratios inside the NPGP are higher than outside the NPGP, with median values ranging between 20 – 10,000 microplastics (500 µm – 5 mm) per individual depending on species [3]. When rescaled to microplastics between 1 µm – 5 mm in size using probability density functions [52–54], median microplastics-to-neuston ratios range between 17,000 – 8,500,000 number of particles per individual. Interactions between neuston and microplastic are therefore considered to be frequent (value = 3, certainty = 3).

Cleanup

Impacts of open ocean cleanups on neuston have been assessed to range from potentially negligible to severe [47,55]. Any neuston larger than the mesh of the cleanup system wings (i.e., > 16 mm) will likely

be retained or impacted. With the exception of *Velella vellela* and some siphonophores, the dominant neuston species in the NPGP are typically < 16 mm [3,26,47,56]. Consequently, *Velella velella* was the only member of the neuston observed in the bycatch throughout 18 cleanup campaigns by The Ocean Cleanup, with a total of ~1,000 individuals collected during one extraction, representing 3.9% and < 0.1% of the total primary incidental catch by count and weight, respectively (Table S4).

However, due to their small size and often gelatinous nature, it is challenging to quantify primary incidental capture of neuston, resulting in possible harm to neuston that is not visible only by investigation of bycatch. For example, neuston species smaller than the mesh of the retention zone (i.e., < 10 mm) could be impacted upon filling of the retention zone with plastics and subsequent clogging of the mesh. Additionally, neuston accumulating in the retention zone could become crushed between the plastics, thus decreasing the likelihood of their detection and identification. We therefore collected additional data by deploying Manta trawls (Ocean Instruments, Inc.) in front and behind The Ocean Cleanup's System 03 during cleanup operations in the NPGP in 2023. These samples were used to investigate potential impacts on density and diversity as a result of the cleanup.

Manta trawls, with a mouth area of 90 cm x 15 cm (width x height) and a square mesh net of 500 µm (333 µm mesh size cod-end), were concurrently deployed from one of the towing vessels, as well as from a Manta trawl hooked onto the system approximately 150 m behind the retention system for 30 min at a towing speed of < 2 knots. Duplicate samples (i.e., two consecutive deployments) were taken each time. A total of 20 Manta trawl samples (10 pairs each in front and behind) were collected between July 31 and November 3, 2023 (Table S8). The Manta trawl samples were subsequently processed and analyzed according to the methodology described in [26].

Our analysis revealed no systematic difference in observed neuston counts for samples collected in front vs behind the cleanup system (Figure S1). *Halobates* spp. and larger species such as *Velella*

velella and siphonophores often depicted lower counts behind the system, yet not consistently. *Janthina janthina* and *Glaucus* spp. showed generally higher counts behind the system compared to in front. *Porpita porpita* showed no clear pattern. These initial results indicate that *Halobates* spp., *Velella velella* and siphonophores could be impacted by interactions with the cleanup system. However, it remains unclear to what extent our results are masked by the extremely patchy distribution of neuston in the NPGP, with densities varying by orders of magnitude over small scales such as on the distance between the Manta trawls deployed from the towing vessel and from behind the cleanup system (i.e., < 2 km) [3,26,47]. We did not detect visible physical injuries on neuston collected behind the cleanup system, indicating limited negative effects on individual organisms. Thus, more data on neuston counts and composition in front vs behind the cleanup system are needed to conclude on impacts. Based on currently available data, and in line with a previous impact assessment for System 03 [47], we estimate the overall cleanup intensity on neuston to be moderate (value = 2, certainty = 3). Given that entrapment is unlikely due to the large mesh of the system (except for *Velella velella* and siphonophores) and that we did not observe any visible injuries for individuals collected behind the cleanup system, the overall impact reversibility is considered at most difficult to reverse (value = 2, certainty = 2). The presence of neuston in the NPGP [3,26,47,56] and its limited mobility (i.e., the ability to move away from the cleanup system) suggests that interactions with neuston during cleanup could be frequent (value = 3, certainty = 2).

Impacts on bony fish

Macroplastics

Given the high contribution of abandoned, lost, or discarded fishing gear (ALDFG) in the NPGP [1,11], entanglement of fish in macroplastics may occur in the NPGP. However, observational data on the entanglement of fish in the NPGP is largely missing [57]. In pelagic environments such as the NPGP, large fish have the capacity to ingest macroplastics [57–59]. Larger fish may also aid in the breakup of

large plastics into smaller fragments, as evidenced through bite marks present on items recovered from the sea surface [60,61], including on items recovered during cleanup operations in the NPGP (Royer et al., unpublished data). Entanglement and ingestion can result in injury or death of the animal, resulting in an estimated moderate impact intensity (value = 2, certainty = 1). Furthermore, floating macroplastics have been shown to facilitate the long-distance dispersal of non-native fish species, thus potentially altering fish biogeography [62]. Although studies are sparse [27], entanglement and changes in fish biogeography are likely at best difficult to reverse (value = 2, certainty = 1). The reported number of fish species potentially affected by entanglement has almost doubled since 1997 [63], but available data [27,63] suggests that the frequency of impact remains low (value = 1, certainty = 3).

Microplastics

The impacts of microplastic ingestion have been shown to range from sub-lethal effects to mortality [27]. Current levels of microplastic (1 µm – 5 mm) pollution in the NPGP (> 84,000 particles/m^3) [1–3] reach estimated hazardous microplastic threshold effect concentrations (HC_5, ~75,600 particles/m^3) for aquatic species [33]. Thus, based on the reported HC_5 and measured microplastics concentrations in the NPGP, we assume a moderate impact intensity of microplastics on fish in the NPGP (value = 2, certainty = 3), but note that impact intensity could increase in the near future due to the rapid increase in microplastics concentrations in the NPGP [2,3].

Concentrations measured on plastics collected from the NPGP often had plastic/lipid fugacity ratios higher than one for many pollutants when compared to concentrations measured in fish from the region [38]. Such positive fugacity ratios indicate the possible transfer of plastic-associated chemicals (additives and adsorbed pollutants) to biota in the NPGP, suggesting that at least some effects of microplastic ingestion could be difficult to reverse (value = 2, certainty = 1). Globally, incidence rates of microplastic ingestion by fish have increased over the last decade, reaching an average of 26% [64]. Thus, microplastic ingestion by fish in the NPGP is considered abundant (value = 3, certainty = 3).

Cleanup

Whenever possible, fish captured during cleanup operations are returned to the water after plastic removal. Most fish extracted with the plastic, however, do not survive the extraction and sorting procedure on deck, resulting in a high impact intensity (value = 3, certainty = 5) and a mostly irreversible impact (value = 3, certainty = 5). In over 18 cleanup campaigns by The Ocean Cleanup, bony fish accounted for 84% and 43% of the total primary incidental catch by count and weight, respectively (Table S4). Species typically known to associate with flotsam or drifting algae, such as blennies (Bleniidae), sargassum fish (*H. histrio*) and Indo-pacific sergeants (*Abudefduf vaigiensis*) made up the majority of incidental catch for fish, together accounting for over 77% of all fish collected during cleanup, in number. In terms of weight, flying fish (*Hirundichthys* spp., 28%), Indo-pacific sergeants (20%) and amberjacks (*Seriola* spp., 16%) were the most representative. Other fish extracted with the plastic debris included at least 18 different species, each representing less than 2% of incidentally captured fish (see Table S4 for a detailed description of all of the fish bycatch). The number of fish captured, however, is unlikely to be significant on the regional or population level for any of the captured fish species [47]. During cleanup operations, numerous fish were observed on underwater cameras swimming into and out of the cleanup system through the mesh and dedicated openings, indicating that many of the fish that entered the cleanup system were not captured due to the slow towing speed (< 2.5 knots) of the system and its design, thus suggesting only occasional capture (value = 2, certainty = 2).

Impacts on elasmobranchs

Macroplastics

Given the high contribution of ALDFG in the NPGP [1,11], entanglement of sharks and rays in macroplastics may occur. Such entanglement is expected to cause substantial injuries or death. Macroplastics can also be ingested, as evidenced by macroplastic items found in the stomachs of

different shark species [65–68] and by visible shark bite marks on some plastic items collected from the NPGP during cleanup operations (Royer et al., unpublished data). The effects of macroplastic ingestions are largely unknown, but it is possible that impacts could range from accumulation of persistent toxic compounds in tissues to sub-lethal effects (e.g., gut blockages) to mortality. We therefore assume a moderate impact intensity (value = 2, certainty = 1) and an impact that is at best difficult to reverse [69] (value = 2, certainty = 1).

Reported evidence for entanglement is typically < 5% for sharks caught in shark control programs [65] or fisheries bycatch [70,71]. Investigated shark species present in the NPGP show varying plastic ingestion frequency of occurrence, ranging from 5% for cookiecutter sharks, to 36% and even 78% for pygmy sharks and blue sharks, respectively, with most ingested plastic fragments larger than 5 mm (Shaw et al., unpublished data). In comparison, the reported ingestion frequency of occurrence in the Mediterranean Sea ranges between 0 – 44% [67]. While entanglement may be rare, macroplastic ingestion by sharks is assumed abundant in the NPGP, resulting in a possible high impact frequency (value = 3, certainty = 1).

Microplastics

The effects of microplastic ingestion on elasmobranchs are largely unknown. Following the assessment for zooplankton and bony fish (see sections above), we assume a moderate impact intensity of microplastics in the NPGP (value = 2, certainty = 1) as levels of microplastics exceed the estimated hazardous threshold effect concentration. Possible leaching of additives and adsorbed chemicals from microplastics and subsequent accumulation within tissues as suggested for fish in the NPGP [38] indicates that at least some effects of microplastic ingestion by elasmobranchs could be difficult to reverse (value = 2, certainty = 1). Microplastic ingestion has been observed in the gastrointestinal tract of sharks in the NPGP (blue, pygmy and cookiecutter sharks; Shaw et al., unpublished data; Van Vulpen et al., unpublished data) and beyond [67,72]. Microplastic could be taken up through trophic transfer, as

demonstrated for whales and dolphins, for example [73–75]. Thus, microplastic ingestion by elasmobranchs in the NPGP is considered abundant (value = 3, certainty = 3).

Cleanup

Elasmobranchs removed together with the plastic during cleanup operations typically do not survive the extraction and sorting on deck, resulting in a high impact intensity (value = 3, certainty = 5) and a largely irreversible impact (value = 3, certainty = 5). Throughout 18 cleanup campaigns, a total of 3,031 elasmobranchs were caught, corresponding to 12% of total primary incidental catch by count and 27% by weight, respectively (Table S4). 99.9% of these records were sharks, with only 2 records of pelagic stingrays. Pygmy and cookiecutter sharks (family Dalatiidae) were the most common species, accounting for over 98% of the primary incidental shark catch in number, and 41% in weight. Blue sharks (*Prionace glauca*) represented 1% of elasmobranch incidental capture by count, but 59% by weight. Additional shark species included three kitefin sharks (*Dalatias licha*). Similar to bony fish, sharks may be able to exit the cleanup system through the dedicated openings in the system's retention zone. For example, some blue sharks were observed to freely swim in and out of the cleanup system unharmed. Impacts are therefore estimated to be occasional (value = 2, certainty = 2).

Impacts on sea turtles

Macroplastics

Subtropical gyres are important development and feeding zones for sea turtles, as individuals may spend several years in these offshore waters during their initial oceanic life stage, as well as during their migrations between breeding and foraging areas as adults [76,77]. The North Pacific subtropical gyre is a known occurrence area for sea turtles, mainly loggerhead (*Caretta caretta*), olive ridley (*Lepidochelys olivacea*), green (*Chelonia mydas*) and leatherback sea turtles (*Dermochelys coriacea*) [78–80]. Entanglement of sea turtles in macroplastics, typically associated with ALDFG, is accompanied by substantial health implications, severe injuries or death [27,63,81]. Ingestion of macroplastics has been

linked to adverse health effects in turtles that typically relate to damage or obstruction of the stomach, intestine, and digestive tract, with some studies reporting that 5 – 35% of deceased sea turtles that ingested plastic likely died due to this ingestion [82]. Interactions with macroplastics are therefore likely associated with high impact intensity (value = 3, certainty = 3). Some individuals can escape from entanglement or expel some of the ingested plastics within one or two months, but the severity of wounds found on escaped individuals [27] and the potential accumulation of persistent toxic compounds in tissues upon plastic ingestion suggests that some impacts are at least difficult to reverse (value = 2, certainty = 1). Macroplastic entanglement and ingestion are widespread and abundant [27,81,83,84], with >50% of pelagic turtles estimated to have ingested plastics [85,86]. The probability of interactions with macroplastics is high for turtles residing in the North Pacific subtropical gyre [86–88], and are therefore considered abundant in the NPGP (value = 3, certainty = 3).

Microplastics

Microplastic ingestion, including the potential accumulation of leaching additives and adsorbed pollutants, can cause dietary dilution, which in turn can result in poor health, reduced growth rates, and reproductive output [89]. These mostly sub-lethal effects [90] indicate a low impact intensity (value = 1, certainty = 1) and that some of the effects could be at least difficult to reverse (value = 2, certainty = 1). Microplastic ingestion by sea turtles has been documented widely and with a high frequency of occurrence, including the indirect ingestion of microplastics through consumption of contaminated prey [84,89–92]. Given the high concentration of microplastics, ingestion by sea turtles (particularly through contaminated prey items) in the NPGP therefore is considered abundant (value = 3, certainty = 3).

Cleanup

During the 2.5 years of operations in the NPGP, The Ocean Cleanup documented 76 encounters with sea turtles (Table S7). Nineteen of these turtles were spotted outside of the system's retention zone, of

which 18 required only a change in navigation or no mitigation to avoid capture, while one was spotted entangled in a ghost net and was rescued by the crew. The animal was safely released, and given its severe entanglement, it is unlikely that it would have survived without interference from the cleanup crew. A total of 57 encounters involved sea turtles inside the retention zone, of which 10 were determined to have been previously deceased (tertiary incidental catch; determined based on external decomposition and internal analysis of organs), and one animal entered heavily entangled in a large ghost net, without the possibility of rescue (secondary bycatch). Of the 47 turtles that entered the system presumably alive, 28 exited through the existing bottom holes or were rescued/extracted alive. Seven turtles were found alive after extraction without being detected beforehand and were released back to sea unharmed. Of these, one had a long piece of rope from its mouth to the cloaca, which was removed by the cleanup crew before release, and one was disentangled from nets, with its wounds/scarring indicating entanglement before entering the system (therefore classified as secondary bycatch). Another animal entered the retention zone heavily entangled in a large ghost net, without the possibility of rescue (also classified as secondary bycatch). A total of 11 turtles were believed to have died due to entrapment in the system, all of which were found to have ingested plastics [93]. Taking into account all live turtles observed, this resulted in a mortality rate of 17% and thus a high impact intensity (value = 3, certainty = 3). While most turtles (i.e., 83%) observed during cleanup were unharmed by the operations (or even rescued from plastic entanglement) the impact is irreversible for turtles deceased in the system. Thus, impacts are considered difficult to reverse (value = 2, certainty = 2). Interactions with turtles are reported to be frequent, with average turtle encounters of around one animal per 100 hours of cleaning up (value = 3, certainty = 3).

Impacts on seabirds

Macroplastics

The migration routes and foraging range of numerous seabirds overlap with the pelagic offshore waters of subtropical gyres [94], putting them at risk of entanglement and plastic ingestion. Many seabird species travel great distances during foraging trips to provision their chicks on islands and coastal rookeries. Thus, plastic ingestion is linked to both trophic and intergenerational transfer of debris [95–97], with seabirds acting as a transfer of plastics from the NPGP to coastal rookeries [98,99]. The NPGP has been identified as a high plastic exposure area for seabirds [94]. Seabirds can both ingest macroplastics and become entangled in them [100–102]. The latter is typically associated with fishing gear [101,103] and results in a high mortality [104]. Without human intervention, entanglement is largely considered irreversible for birds [27]. The effects of macroplastic ingestion on bird health are species-specific and dependent on bird physiology, ability to regurgitate, and respective feeding strategies [27,105]. In some cases, macroplastic ingestion is believed to contribute to sub-lethal impacts, including lesions, increased concentrations of plastic-associated chemicals (e.g. trace elements, persistent organic pollutants), disruption of fatty acid composition, starvation, disease, and endoparasite burden [27,38,106–109]. However, ingestion has also been linked to direct mortality due to starvation or blockages and damages to the gastrointestinal tract [109,110]. Thus, the impact intensity of macroplastics (including both entanglement and ingestion) is rated as high (value = 3, certainty = 4), with effects often being irreversible (value = 3, certainty = 3).

Available literature indicates that entanglement probability varies spatially and temporarily, but that it is generally considered low [27]. Ingestion of macroplastics, however, is abundant [95,111,112], and seabirds have been used as biomonitors of plastic pollution (including in the gyres) due to their high susceptibility to ingest plastics [113–117]. Interactions with macroplastics are therefore considered abundant in the NPGP (value = 3, certainty = 3).

Microplastics

Ingestion of microplastics by seabirds can cause sub-lethal effects [27,106,109,118–120]. Impact intensity is therefore considered as low (value = 1, certainty = 3). Concentrations of pollutants measured on plastics

collected from the NPGP often had plastic/lipid fugacity ratios >1 for many pollutants when compared to concentrations measured in seabirds from the region [38]. Such positive fugacity ratios indicate the possible transfer of plastic-associated chemicals (additives and adsorbed pollutants) to seabirds in the NPGP. Although it remains uncertain whether such a transfer occurs [121–124], some effects could at least be difficult to reverse (value = 2, certainty = 1). The NPGP represents a high exposure risk area for seabirds [94], and reports of microplastic ingestion in the area are abundant [94,112,115] (value = 3, certainty = 4).

Cleanup

Between July 2021 and December 2022, 3,213 individual seabirds were observed by protected species observers onboard the cleanup vessels (all records shown in Table S7). Between March and November 2023, only seabirds found on the vessel were reported, corresponding to 47 individual animals (Table S7). Laysan albatross (*Phoebastria immutabilis*), unidentified gulls, and red phalarope (*Phalaropus fulicarius*) were the most common species observed with 709, 665, and 459 individuals, respectively. Most records were of seabirds flying or floating on the water close to the vessels (94%), with only some observed sitting on the cleanup system (0.1%) or otherwise interacting with the vessels (resting or found deceased on deck, 6%). No negative interactions with seabirds and the cleanup system have been recorded throughout all cleanup campaigns by The Ocean Cleanup thus far, with only a few fatal vessel strikes [47]. Resting on the vessels could further provide a benefit to some species. Cleanup impacts are therefore rated as low in intensity (value = 1, certainty = 3). Effects are further considered to be reversible (value = 1, certainty = 2) and interactions to be rare (value = 1, certainty = 3).

<u>Impacts on marine mammals</u>

Macroplastics

Cetaceans are the most common marine mammals present in the NPGP (Table S7). Ingestion of macroplastics by cetaceans and entanglement in macroplastics, particularly ALDFG, is widely reported

[125–128]. Impacts of macroplastic entanglement have been shown to range from loose, to substantial scarring due to cutting into the animal’s tissue and death by drowning. Thus, impact intensity is assumed to be moderate (value = 2, certainty = 3). Observed scarring on marine mammals indicates that some individuals can escape from entanglement, but the depth and severity of wounds suggest that entanglement can be difficult to reverse [27] (value = 2, certainty = 1). Given the high contribution of ALDFG in the NPGP, interactions with macroplastics are considered abundant (value = 3, certainty = 1).

Microplastics

Ingestion of microplastics by cetaceans, either through direct ingestion but mostly by trophic transfer, has been widely reported [45,73–75,129–131]. Although little is known about the health consequences of microplastic ingestion and chemical accumulation in wild vertebrates [132,133], sub-lethal health effects due to the potential accumulation of toxic chemicals or the translocation of microplastics [134] are possible (value = 1, certainty = 1). The presence of microplastics in whale feces [131] indicates that some of the ingested microplastics are egested again, but some effects such as translocation of microplastics could be at least difficult to reverse (value = 2, certainty = 1). Microplastics ingestion rates and loads in baleen whales can reach millions of particles per day in the eastern North Pacific Ocean [75]. Although microplastic ingestion by baleen whales in the NPGP could be lower for animals mainly migrating through the region, microplastic uptake could remain high for toothed whales feeding in the NPGP due to indirect uptake through prey items. Overall, microplastic ingestion by cetaceans in the NPGP is assumed to be abundant (value = 3, certainty = 1).

Cleanup

During all operations by The Ocean Cleanup (including vessel transit between Victoria (British Columbia) and the NPGP, as well as cleanup operations in the NPGP), protected species observers continuously monitor for the presence of marine mammals throughout the day and night. Throughout

18 cleanup campaigns between July 2021 and November 2023, a total of 3,008 individuals were observed, with 96% of these sightings occurring during transit (Table S7). Out of the total number of individuals, 691 were mysticete whales, 2,263 were odontocetes, 42 were pinnipeds (only observed during transit through more coastal waters), and 12 were unidentified cetaceans (information on groups/species detailed in Table S7). No negative impacts were observed, as all individuals swam away on their own unharmed. Impacts during vessel transit to and from land to the NPGP are unlikely due to implemented mitigation measures such as onboard protected species observers, slow transit speed (<14 knots) and use of high-frequency acoustic pingers. Thus, impacts on marine mammals are considered low in intensity (value = 1, certainty = 4), with effects being reversible (value = 1, certainty = 4), and interactions being rare (value = 1, certainty = 4).

Impacts on cephalopods

Macroplastics

Little is known about interactions between macroplastics and pelagic cephalopods, but available data from benthic octopuses shows that macroplastics can be used as shelter, thus providing an advantage for these animals, allowing them to occupy and spawn in areas with limited natural shelter [135]. Although sheltering and laying eggs on macroplastics may result in exposure to potentially toxic chemical additives and adsorbed contaminants, overall impacts are assumed to be mostly beneficial. Consequently, we attribute a low total vulnerability score of 1 for macroplastics impacts on cephalopods, but with low confidence (uncertainty score = 1.0).

Microplastics

Ingestion of microplastics has been documented for both pelagic as well as benthic cephalopods [135]. The health effects of such ingestion are largely unknown, but could potentially include blockages in the digestive tract [136] and behavioral changes [137]. Plastic afloat in the NPGP further can contain high levels of toxic persistent chemicals [38,119], thus posing a potential for transferring associated toxic

compounds to the tissues [37,38,138]. However, it remains unclear to what extent such a transfer of adsorbed chemicals and leaching of plastic additives increases the exposure and thus risk of toxic persistent chemicals [139,140]. We therefore assume a low impact intensity (value = 1, certainty = 1). The potential accumulation of persistent toxic compounds within pelagic squid [138] indicates that at least some effects of microplastic ingestion could be difficult to reverse (value = 2, certainty = 1). The frequency of occurrence of microplastic ingestion by cephalopods in the open ocean remains unknown due to the scarcity of data [135,136,138]. Here, we therefore conservatively assume a medium score for impact frequency (value = 2, certainty = 1).

Cleanup

Incidental capture of cephalopods results in high mortality, and thus a high impact intensity (value = 3, certainty = 4) that is irreversible (value = 3, certainty = 5). Throughout 18 cleanup campaigns by The Ocean Cleanup between July 2021 and November 2023, cephalopods accounted for 0.3% and 2.7% of the total primary incidental catch by count and weight, respectively (Table S4), suggesting a low impact frequency (value = 1, certainty = 3).

<u>Impacts on rafting species</u>

Macroplastics

In the NPGP, floating macroplastics provide a substrate for growth [25] and oviposition [141]. Invertebrate biofouling was observed to be present on 98% of the plastic debris afloat in the NPGP and included Bryozoa, Crustacea, Chelicerata, and Cnidaria, with most taxa (80%) depicting a coastal origin [25]. Thus, a vast majority of these rafting organisms are not native to the open ocean, and plastic pollution sustains their persistence (i.e., survival and reproduction) in the open ocean [25]. While the impacts of exposure to plastic-associated chemicals [142] are largely unknown, macroplastic pollution seems beneficial for rafting species overall. Consequently, we attribute a low total vulnerability score of 1 for macroplastic impacts on rafting species, with medium confidence (uncertainty score = 0.33).

Microplastics

Rafting species in the NPGP are exposed to high levels of microplastic pollution in the NPGP and have been shown to ingest microplastics [143], which could result in sub-lethal effects (value = 1, certainty = 1). The possible accumulation of persistent chemicals indicates that some impacts could be at least difficult to reverse (value = 2, certainty = 1). Microplastics were found in around 34% of barnacles rafting on macroplastics in the NPGP [143], indicating that microplastic ingestion by rafting species in the NPGP could be abundant (value = 3, certainty = 1).

Cleanup

With the exception of some organisms such as crabs, which can be released back to the water after plastic removal from the ocean, most organisms sessile on the plastic debris (considered as secondary incidental catch) will be removed with the plastics, resulting in high mortality. Overall, the impact intensity is therefore rated high (value = 3, certainty = 5), and effects are considered irreversible (value = 3, certainty = 5) due to the high mortality and direct association with the plastics. Invertebrate biofouling on plastic debris afloat in the NPGP is common [25]. As a result, invertebrates (particularly crustaceans) dominate the secondary incidental catch observed by The Ocean Cleanup throughout their 18 cleanup campaigns between July 2021 and November 2023 (Table S5). Considering that rafting organisms are frequently present on plastic debris afloat in the NPGP [25], interactions between cleanup operations and rafting species are rated as abundant (value = 3, certainty = 4).

References

1. Lebreton, L. *et al.* Evidence that the Great Pacific Garbage Patch is rapidly accumulating plastic. *Sci Rep* **8**, 1–15 (2018).

2. Lebreton, L. *et al.* Seven years into the North Pacific Garbage Patch: legacy plastic fragments rising disproportionally faster than larger floating objects. *under review* (2024).

3. Egger, M. *et al.* Densities of neuston often not elevated within plastic hotspots territory inside the North Pacific Garbage Patch. *Environmental Research Ecology* **3**, (2024).

4. Maximenko, N., Hafner, J., Kamachi, M. & MacFadyen, A. Numerical simulations of debris drift from the Great Japan Tsunami of 2011 and their verification with observational reports. *Mar Pollut Bull* **132**, 5–25 (2018).

5. Carlton, J. T. *et al.* Tsunami-driven rafting: Transoceanic species dispersal and implications for marine biogeography. *Science (1979)* **357**, 1402–1406 (2017).

6. Friedlander, A. *et al. The State of Coral Reef Ecosystems of the Northwestern Hawaiian Islands*. *NO* (2005).

7. Brignac, K. C. *et al.* Marine Debris Polymers on Main Hawaiian Island Beaches, Sea Surface, and Seafloor. *Environ Sci Technol* (2019) doi:10.1021/acs.est.9b03561.

8. Egger, M., Sulu-Gambari, F. & Lebreton, L. First evidence of plastic fallout from the North Pacific Garbage Patch. *Sci Rep* **10**, 7495 (2020).

9. Zhao, S., Mincer, T. J., Lebreton, L. & Egger, M. Pelagic microplastics in the North Pacific subtropical gyre: a prevalent anthropogenic component of the particulate organic carbon pool. *PNAS Nexus* (2023) doi:10.1093/pnasnexus/pgad070.

10. Lebreton, L., Egger, M. & Slat, B. A global mass budget for positively buoyant macroplastic debris in the ocean. *Sci Rep* **9**, 1700782 (2019).

11. Lebreton, L. *et al.* Industrialised fishing nations largely contribute to floating plastic pollution in the North Pacific subtropical gyre. *Sci Rep* **12**, (2022).

12. Delre, A. *et al.* Plastic photodegradation under simulated marine conditions. *Mar Pollut Bull* **187**, (2023).

13. Egger, M. *et al.* A spatially variable scarcity of floating microplastics in the eastern North Pacific Ocean. *Environmental Research Letters* **15**, 114056 (2020).

14. Lobelle, D. *et al.* Global modeled sinking characteristics of biofouled microplastic. *J Geophys Res Oceans* 1–15 (2021) doi:10.1029/2020jc017098.

15. Kvale, K., Prowe, A. E. F., Chien, C. T., Landolfi, A. & Oschlies, A. The global biological microplastic particle sink. *Sci Rep* **10**, 1–12 (2020).

16. Galgani, L. *et al.* Hitchhiking into the Deep: How Microplastic Particles are Exported through the Biological Carbon Pump in the North Atlantic Ocean. *Environ Sci Technol* **56**, 15638–15649 (2022).

17. Richon, C., Kvale, K., Lebreton, L. & Egger, M. Legacy oceanic plastic pollution must be addressed to mitigate possible long-term ecological impacts. *Microplastics and Nanoplastics* **3**, 25 (2023).

18. Eriksen, M. *et al.* Plastic pollution in the world's cceans: more than 5 trillion plastic pieces weighing over 250,000 tons afloat at sea. *PLoS One* **9**, e111913 (2014).

19. van Sebille, E. *et al.* A global inventory of small floating plastic debris. *Environmental Research Letters* **10**, 124006 (2015).

20. Kaandorp, M. L. A., Lobelle, D., Kehl, C., Dijkstra, H. A. & van Sebille, E. Global mass of buoyant marine plastics dominated by large long-lived debris. *Nat Geosci* **16**, 689–694 (2023).

21. Rynek, R. *et al.* Hotspots of Floating Plastic Particles across the North Pacific Ocean. *Environ Sci Technol* (2024) doi:10.1021/acs.est.3c05039.

22. Law, K. L. *et al.* Distribution of surface plastic debris in the eastern pacific ocean from an 11-year data set. *Environ Sci Technol* **48**, 4732–4738 (2014).

23. de Vries, R., Egger, M., Mani, T. & Lebreton, L. Quantifying floating plastic debris at sea using vessel-based optical data and artificial intelligence. *Remote Sens (Basel)* **13**, 1–16 (2021).

24. Goldstein, M. C., Titmus, A. J. & Ford, M. Scales of spatial heterogeneity of plastic marine debris in the northeast Pacific Ocean. *PLoS One* **8**, (2013).

25. Haram, L. E. *et al.* Extent and reproduction of coastal species on plastic debris in the North Pacific Subtropical Gyre. *Nat Ecol Evol* (2023) doi:10.1038/s41559-023-01997-y.

26. Egger, M. *et al.* Relative Abundance of Floating Plastic Debris and Neuston in the Eastern North Pacific Ocean. *Front Mar Sci* **8**, 1–13 (2021).

27. Beaumont, N. J. *et al.* Global ecological, social and economic impacts of marine plastic. *Mar Pollut Bull* **142**, 189–195 (2019).

28. Cole, M., Lindeque, P., Fileman, E., Halsband, C. & Galloway, T. S. The impact of polystyrene microplastics on feeding, function and fecundity in the marine copepod Calanus helgolandicus. *Environ Sci Technol* **49**, 1130–1137 (2015).

29. Cole, M. *et al.* Effects of Nylon Microplastic on Feeding, Lipid Accumulation, and Moulting in a Coldwater Copepod. *Environ Sci Technol* **53**, 7075–7082 (2019).

30. Cole, M. *et al.* Microplastic ingestion by zooplankton. *Environ Sci Technol* **47**, 6646–6655 (2013).

31. Bejgarn, S., MacLeod, M., Bogdal, C. & Breitholtz, M. Toxicity of leachate from weathering plastics: An exploratory screening study with Nitocra spinipes. *Chemosphere* **132**, 114–119 (2015).

32. Focardi, A. *et al.* Plastic leachates impair picophytoplankton and dramatically reshape the marine microbiome. *Microbiome* **10**, (2022).

33. Koelmans, A. A. *et al.* Risk assessment of microplastic particles. *Nat Rev Mater* **7**, 138–152 (2022).

34. Brandon, J., Goldstein, M. & Ohman, M. D. Long-term aging and degradation of microplastic particles: Comparing in situ oceanic and experimental weathering patterns. *Mar Pollut Bull* **110**, 299–308 (2016).

35. Rios Mendoza, L. M. & Jones, P. R. Characterisation of microplastics and toxic chemicals extracted from microplastic samples from the North Pacific Gyre. *Environmental Chemistry* **12**, 611–617 (2015).

36. Rios, L. M., Jones, P. R., Moore, C. & Narayan, U. V. Quantitation of persistent organic pollutants adsorbed on plastic debris from the Northern Pacific Gyre's 'eastern garbage patch'. *Journal of Environmental Monitoring* **12**, 2226–2236 (2010).

37. Gassel, M., Harwani, S., Park, J. S. & Jahn, A. Detection of nonylphenol and persistent organic pollutants in fish from the North Pacific Central Gyre. *Mar Pollut Bull* **73**, 231–242 (2013).

38. Chen, Q. *et al.* Pollutants in Plastics within the North Pacific Subtropical Gyre. *Environ Sci Technol* **52**, 446–456 (2018).

39. Yu, S.-P., Cole, M. & Chan, B. K. K. Effects of microplastic on zooplankton survival and sublethal responses. in *Oceanography and Marine Biology: An Annual Review* (eds. Hawkins, S. J. , et al.) vol. 58 351–393 (CRC Press, 2020).

40. Botterell, Z. L. R. *et al.* Bioavailability and effects of microplastics on marine zooplankton: A review. *Environmental Pollution* **245**, 98–110 (2019).

41. Setälä, O., Fleming-Lehtinen, V. & Lehtiniemi, M. Ingestion and transfer of microplastics in the planktonic food web. *Environmental Pollution* **185**, 77–83 (2014).

42. Richon, C., Gorgues, T., Paul-Pont, I. & Maes, C. Zooplankton exposure to microplastics at global scale: Influence of vertical distribution and seasonality. *Front Mar Sci* **9**, (2022).

43. Everaert, G. *et al.* Risks of floating microplastic in the global ocean. *Environmental Pollution* **267**, (2020).

44. Desforges, J. P. W., Galbraith, M. & Ross, P. S. Ingestion of Microplastics by Zooplankton in the Northeast Pacific Ocean. *Arch Environ Contam Toxicol* **69**, 320–330 (2015).

45. Torres, L. G. *et al.* Zoop to poop: assessment of microparticle loads in gray whale zooplankton prey and fecal matter reveal high daily consumption rates. *Front Mar Sci* **10**, (2023).

46. Brandon, J. A., Freibott, A. & Sala, L. M. Patterns of suspended and salp-ingested microplastic debris in the North Pacific investigated with epifluorescence microscopy. *Limnol Oceanogr Lett* **5**, 46–53 (2020).

47. Ocean Sciences Inc., C. *The Ocean Cleanup Final S03 Environmental Impact Assessment*. https://assets.theoceancleanup.com/app/uploads/2023/08/System-03-Environmental-Impact-Assessment-The-Ocean-Cleanup.pdf (2023).

48. Gregory, M. R. Environmental implications of plastic debris in marine settings- entanglement, ingestion, smothering, hangers-on, hitch-hiking and alien invasions. *Philosophical Transactions of the Royal Society B: Biological Sciences* **364**, 2013–2025 (2009).

49. Goldstein, M. C., Carson, H. S. & Eriksen, M. Relationship of diversity and habitat area in North Pacific plastic-associated rafting communities. *Mar Biol* **161**, 1441–1453 (2014).

50. Bryant, J. A. *et al.* Diversity and Activity of Communities Inhabiting Plastic Debris in the North Pacific Gyre. *mSystems* **1**, 1–19 (2016).

51. Betti, F. *et al.* Massive strandings of Velella velella (Hydrozoa: Anthoathecata: Porpitidae) in the Ligurian Sea (North-western Mediterranean Sea). *European Zoological Journal* **86**, 343–353 (2019).

52. Kooi, M. & Koelmans, A. A. Simplifying Microplastic via Continuous Probability Distributions for Size, Shape,and Density. *Environ Sci Technol Lett* **6**, 551–557 (2019).

53. Koelmans, A. A., Redondo-Hasselerharm, P. E., Mohamed Nor, N. H. & Kooi, M. Solving the Nonalignment of Methods and Approaches Used in Microplastic Research to Consistently Characterize Risk. *Environ Sci Technol* **54**, 12307–12315 (2020).

54. Kooi, M. *et al.* Characterizing the multidimensionality of microplastics across environmental compartments. *Water Res* **202**, (2021).

55. Spencer, M. *et al.* Estimating the impact of new high seas activities on the environment: the effects of ocean-surface macroplastic removal on sea surface ecosystems. *PeerJ* **11**, e15021 (2023).

56. Chong, F. *et al.* High concentrations of floating neustonic life in the plastic-rich North Pacific Garbage Patch. *PLoS Biol* **21**, e3001646 (2023).

57. Santos de Moura, M. & Vianna, M. A new threat: assessing the main interactions between marine fish and plastic debris from a scientometric perspective. *Rev Fish Biol Fish* **30**, 623–636 (2020).

58. Neto, J. G. B. *et al.* Ingestion of plastic debris by commercially important marine fish in southeast-south Brazil. *Environmental Pollution* **267**, (2020).

59. Yick, J. L. & Travers, T. The ingestion of large plastics by recreationally caught southern bluefin tuna Thunnus maccoyii off southern Australia. *Mar Pollut Bull* **175**, (2022).

60. Carson, H. S. The incidence of plastic ingestion by fishes: From the prey's perspective. *Mar Pollut Bull* **74**, 170–174 (2013).

61. Po, B. H. K., Lo, H. S., Cheung, S. G. & Lai, K. P. Characterisation of an unexplored group of microplastics from the South China Sea: Can they be caused by macrofaunal fragmentation? *Mar Pollut Bull* **155**, (2020).

62. Benadon, C. *et al.* Marine debris facilitates the long-distance dispersal of fish species. *Mar Biol* **171**, (2024).

63. Gall, S. C. & Thompson, R. C. The impact of debris on marine life. *Mar Pollut Bull* **92**, 170–179 (2015).

64. Savoca, M. S., Mcinturf, A. G. & Hazen, E. L. Plastic ingestion by marine fish is widespread and increasing. 1–12 (2021) doi:10.1111/gcb.15533.

65. Cliff, G., Dudley, S. F. J., Ryan, P. G. & Singleton, N. Large sharks and plastic debris in KwaZulu-Natal, South Africa. in *Marine and Freshwater Research* vol. 53 575–581 (2002).

66. Abreo, N. A. S., Blatchley, D. & Superio, M. D. Stranded whale shark (Rhincodon typus) reveals vulnerability of filter-feeding elasmobranchs to marine litter in the Philippines. *Mar Pollut Bull* **141**, 79–83 (2019).

67. Bernardini, I., Garibaldi, F., Canesi, L., Fossi, M. C. & Baini, M. First data on plastic ingestion by blue sharks (Prionace glauca) from the Ligurian Sea (North-Western Mediterranean Sea). *Mar Pollut Bull* **135**, 303–310 (2018).

68. Barreto, R. *et al.* Macro-debris ingestion and entanglement by blue sharks (Prionace glauca Linnaeus, 1758) in the temperate South Atlantic Ocean. *Mar Pollut Bull* **145**, 214–218 (2019).

69. Wegner, N. C. & Cartamil, D. P. Effects of prolonged entanglement in discarded fishing gear with substantive biofouling on the health and behavior of an adult shortfin mako shark, Isurus oxyrinchus. *Mar Pollut Bull* **64**, 391–394 (2012).

70. Sazima, I., Gadig, O. B. F., Namora, R. C. & Motta, S. Plastic debris collars on juvenile carcharhinid sharks (Rhizoprionodon lalandii) in southwest Atlantic. *Mar Pollut Bull* **44**, 1147–1149 (2002).

71. Mucientes, G. & Queiroz, N. Presence of plastic debris and retained fishing hooks in oceanic sharks. *Mar Pollut Bull* **143**, 6–11 (2019).

72. Munno, K. *et al.* High microplastic and anthropogenic particle contamination in the gastrointestinal tracts of tiger sharks (Galeocerdo cuvier) caught in the western North Atlantic Ocean. *Environmental Pollution* **344**, (2024).

73. Burkhardt-Holm, P. & N’Guyen, A. Ingestion of microplastics by fish and other prey organisms of cetaceans, exemplified for two large baleen whale species. *Mar Pollut Bull* **144**, 224–234 (2019).

74. Dool, T. & Bosker, T. Predicted microplastic uptake through trophic transfer by the short-beaked common dolphin (Delphinus delphis) and common bottlenose dolphin (Tursiops truncatus) in the Northeast Atlantic Ocean and Mediterranean Sea. *Mar Pollut Bull* **180**, (2022).

75. Kahane-Rapport, S. R. *et al.* Field measurements reveal exposure risk to microplastic ingestion by filter-feeding megafauna. *Nat Commun* **13**, (2022).

76. Hays, G. C. & Scott, R. Global patterns for upper ceilings on migration distance in sea turtles and comparisons with fish, birds and mammals. *Funct Ecol* **27**, 748–756 (2013).

77. Bolten, A. B. Variation in Sea Turtle Life History Patterns: Neritic vs. Oceanic Developmental Stages. in *The Biology of Sea Turtles* (eds. Lutz, P. L., Musick, J. A. & Wyneken, J.) 243–258 (CRC Press, 2003).

78. Polovina, J. J. *et al.* Forage and migration habitat of loggerhead (Caretta caretta) and olive ridley (Lepidochelys olivacea) sea turtles in the central North Pacific Ocean. *Fish Oceanogr* **13**, 36–51 (2004).

79. Parker, D. M., Dutton, P. H. & Balazs, G. H. Oceanic diet and distribution of haplotypes for the green turtle, chelonia mydas, in the Central North Pacific. *Pac Sci* **65**, 419–431 (2011).

80. Roe, J. H. *et al.* Predicting bycatch hotspots for endangered leatherback turtles on longlines in the Pacific Ocean. *Proceedings of the Royal Society B: Biological Sciences* **281**, (2014).

81. Duncan, E. M. *et al.* A global review of marine turtle entanglement in anthropogenic debris: A baseline for further action. *Endanger Species Res* **34**, 431–448 (2017).

82. Schuyler, Q., Hardesty, B. D., Wilcox, C. & Townsend, K. Global Analysis of Anthropogenic Debris Ingestion by Sea Turtles. *Conservation Biology* **28**, 129–139 (2014).

83. Garrard, S. L. *et al.* Identifying potential high-risk zones for land-derived plastic litter to marine megafauna and key habitats within the North Atlantic. *Science of The Total Environment* **922**, 171282 (2024).

84. Rodríguez, Y. *et al.* Litter ingestion and entanglement in green turtles: An analysis of two decades of stranding events in the NE Atlantic. *Environmental Pollution* **298**, (2022).

85. Schuyler, Q., Hardesty, B. D., Wilcox, C. & Townsend, K. To eat or not to eat? debris selectivity by marine turtles. *PLoS One* **7**, (2012).

86. Schuyler, Q. A. *et al.* Risk analysis reveals global hotspots for marine debris ingestion by sea turtles. *Glob Chang Biol* **22**, 567–576 (2016).

87. Høiberg, M. A., Woods, J. S. & Verones, F. Global distribution of potential impact hotspots for marine plastic debris entanglement. *Ecol Indic* **135**, (2022).

88. Wedemeyer-Strombel, K. R. *et al.* High frequency of occurrence of anthropogenic debris ingestion by sea turtles in the North Pacific Ocean. *Mar Biol* **162**, 2079–2091 (2015).

89. Nelms, S. E. *et al.* Plastic and marine turtles: A review and call for research. *ICES Journal of Marine Science* **73**, 165–181 (2016).

90. Clukey, K. E., Lepczyk, C. A., Balazs, G. H., Work, T. M. & Lynch, J. M. Investigation of plastic debris ingestion by four species of sea turtles collected as bycatch in pelagic Pacific longline fisheries. *Mar Pollut Bull* **120**, 117–125 (2017).

91. Choi, D. Y., Gredzens, C. & Shaver, D. J. Plastic ingestion by green turtles (Chelonia mydas) over 33 years along the coast of Texas, USA. *Mar Pollut Bull* **173**, (2021).

92. Petry, M. V., Araújo, L. D., Brum, A. C., Benemann, V. R. F. & Finger, J. V. G. Plastic ingestion by juvenile green turtles (Chelonia mydas) off the coast of Southern Brazil. *Mar Pollut Bull* **167**, (2021).

93. The Ocean Cleanup. System 002 and marine life: Prevention and mitigation. *https://theoceancleanup.com/updates/system-002-and-marine-life-prevention-and-mitigation/* (2023).

94. Clark, B. L. *et al.* Global assessment of marine plastic exposure risk for oceanic birds. *Nat Commun* **14**, 3665 (2023).

95. Young, L. C., Vanderlip, C., Duffy, D. C., Afanasyev, V. & Shaffer, S. A. Bringing home the trash: Do colony-based differences in foraging distribution lead to increased plastic ingestion in Laysan albatrosses? *PLoS One* **4**, (2009).

96. Spear, L. B., Ainley, D. G. & Ribic, C. A. Incidence of plastic in seabirds from the tropical pacific, 1984-1991: Relation with distribution of species, sex, age, season, year and body weight. *Mar Environ Res* **40**, 123–146 (1995).

97. Carey, M. J. Intergenerational transfer of plastic debris by Short-tailed Shearwaters (Ardenna tenuirostris). *Emu* **111**, 229–234 (2011).

98. Provencher, J. F., Vermaire, J. C., Avery-Gomm, S., Braune, B. M. & Mallory, M. L. Garbage in guano? Microplastic debris found in faecal precursors of seabirds known to ingest plastics. *Science of the Total Environment* **644**, 1477–1484 (2018).

99. Grant, M. L., Lavers, J. L., Hutton, I. & Bond, A. L. Seabird breeding islands as sinks for marine plastic debris. *Environmental Pollution* **276**, (2021).

100. Roman, L., Hardesty, B. D., Hindell, M. A. & Wilcox, C. Disentangling the influence of taxa, behaviour and debris ingestion on seabird mortality. *Environmental Research Letters* **15**, (2020).

101. Ryan, P. G. Entanglement of birds in plastics and other synthetic materials. *Mar Pollut Bull* **135**, 159–164 (2018).

102. Hyrenbach, K. D. *et al.* First evidence of plastic ingestion by white-tailed tropicbirds from O'Ahu, Hawai'I. *Mar Ornithol* **41**, 167–169 (2014).

103. Costa, R. A. *et al.* Prevalence of entanglements of seabirds in marine debris in the central Portuguese coast. *Mar Pollut Bull* **161**, (2020).

104. Votier, S. C., Archibald, K., Morgan, G. & Morgan, L. The use of plastic debris as nesting material by a colonial seabird and associated entanglement mortality. *Mar Pollut Bull* **62**, 168–172 (2011).

105. Tavares, D. C., de Moura, J. F., Merico, A. & Siciliano, S. Incidence of marine debris in seabirds feeding at different water depths. *Mar Pollut Bull* **119**, 68–73 (2017).

106. Ryan, P. G., Connell, A. D. & Gardner, B. D. Marine Pollution Bulletin Plastic Ingestion and PCBs in Seabirds: Is There a Relationship ? *Mar Pollut Bull* **19**, 174–176 (1988).

107. Lopes, C. S. *et al.* Fatty acids composition in yellow-legged (Larus michahellis) and lesser black-backed (Larus fuscus) gulls from natural and urban habitats in relation to the ingestion of anthropogenic materials. *Science of the Total Environment* **809**, (2022).

108. Puskic, P. S. *et al.* Uncovering the sub-lethal impacts of plastic ingestion by shearwaters using fatty acid analysis. *Conserv Physiol* **7**, (2019).

109. Puskic, P. S. *et al.* Exploring the pathology of liver, kidney, muscle, and stomach of fledgling seabirds associated with plastic ingestion. *J Hazard Mater* **465**, 133306 (2024).

110. Pierce, K. E., Harris, R. J., Larned, L. S. & Pokras, M. A. Obstruction and starvation associated with plastic ingestion in northern gannet Morus Basanus and a greater shearwater Puffinus Gravis. *Mar Ornithol* **32**, 187–189 (2004).

111. Vlietstra, L. S. & Parga, J. A. Long-term changes in the type, but not amount, of ingested plastic particles in short-tailed shearwaters in the southeastern Bering Sea. *Mar Pollut Bull* **44**, 945–955 (2002).

112. Blight, L. K. & Burger, A. E. Occurrence of plastic particles in seabirds from the eastern North Pacific. *Mar Pollut Bull* **34**, 323–325 (1997).

113. Van Franeker, J. A. & Law, K. L. Seabirds, gyres and global trends in plastic pollution. *Environmental Pollution* **203**, 89–96 (2015).

114. Van Franeker, J. A. *et al.* Monitoring plastic ingestion by the northern fulmar Fulmarus glacialis in the North Sea. *Environmental Pollution* **159**, 2609–2615 (2011).

115. Avery-Gomm, S. *et al.* Northern fulmars as biological monitors of trends of plastic pollution in the eastern North Pacific. *Mar Pollut Bull* **64**, 1776–1781 (2012).

116. Savoca, M. S. *et al.* Towards a North Pacific Ocean long-term monitoring program for plastic pollution: A review and recommendations for plastic ingestion bioindicators. *Environmental Pollution* **310**, (2022).

117. Rodríguez, Y. *et al.* Cory’s shearwater as a key bioindicator for monitoring floating plastics. *Environ Int* **186**, (2024).

118. McCann Smith, E., Bartosova, Z., Wagner, M., Jaspers, V. L. B. & Monclús, L. Exposure to microplastics affects fatty acid composition in the Japanese quail depending on sex and particle size. *Science of the Total Environment* **912**, (2024).

119. Chu, S. *et al.* Perfluoroalkyl sulfonates and carboxylic acids in liver, muscle and adipose tissues of black-footed albatross (Phoebastria nigripes) from Midway Island, North Pacific Ocean. *Chemosphere* **138**, 60–66 (2015).

120. Kühn, S., Booth, A. M., Sørensen, L., van Oyen, A. & van Franeker, J. A. Transfer of Additive Chemicals From Marine Plastic Debris to the Stomach Oil of Northern Fulmars. *Front Environ Sci* **8**, 1–14 (2020).

121. Collard, F. *et al.* Can plastic related chemicals be indicators of plastic ingestion in an Arctic seabird? *Chemosphere* **355**, (2024).

122. Tanaka, K. *et al.* In Vivo Accumulation of Plastic-Derived Chemicals into Seabird Tissues. *Current Biology* **30**, 723-728.e3 (2020).

123. Tanaka, K. *et al.* Facilitated Leaching of Additive-Derived PBDEs from Plastic by Seabirds' Stomach Oil and Accumulation in Tissues. *Environ Sci Technol* **49**, 11799–11807 (2015).

124. Coffin, S., Huang, G. Y., Lee, I. & Schlenk, D. Fish and Seabird Gut Conditions Enhance Desorption of Estrogenic Chemicals from Commonly-Ingested Plastic Items. *Environ Sci Technol* **53**, 4588–4599 (2019).

125. Solomando, A., Pujol, F., Sureda, A. & Pinya, S. Evaluating the Presence of Marine Litter in Cetaceans Stranded in the Balearic Islands (Western Mediterranean Sea). *Biology (Basel)* **11**, (2022).

126. Simmonds, M. P. Cetaceans and Marine Debris: The Great Unknown. *J Mar Biol* **2012**, 1–8 (2012).

127. Kühn, S. & van Franeker, J. A. Quantitative overview of marine debris ingested by marine megafauna. *Mar Pollut Bull* **151**, (2020).

128. Jacobsen, J. K., Massey, L. & Gulland, F. Fatal ingestion of floating net debris by two sperm whales (Physeter macrocephalus). *Mar Pollut Bull* **60**, 765–767 (2010).

129. Alava, J. J. Modeling the Bioaccumulation and Biomagnification Potential of Microplastics in a Cetacean Foodweb of the Northeastern Pacific: A Prospective Tool to Assess the Risk Exposure to Plastic Particles. *Front Mar Sci* **7**, (2020).

130. Zantis, L. J., Carroll, E. L., Nelms, S. E. & Bosker, T. Marine mammals and microplastics: A systematic review and call for standardisation. *Environmental Pollution* **269**, (2021).

131. Zantis, L. J. *et al.* Assessing microplastic exposure of large marine filter-feeders. *Science of the Total Environment* **818**, (2022).

132. de Sá, L. C., Oliveira, M., Ribeiro, F., Rocha, T. L. & Futter, M. N. Studies of the effects of microplastics on aquatic organisms: What do we know and where should we focus our efforts in the future? *Science of the Total Environment* **645**, 1029–1039 (2018).

133. Puskic, P. S., Lavers, J. L. & Bond, A. L. A critical review of harm associated with plastic ingestion on vertebrates. *Science of the Total Environment* **743**, (2020).

134. Merrill, G. B., Hermabessiere, L., Rochman, C. M. & Nowacek, D. P. Microplastics in marine mammal blubber, melon, & other tissues: Evidence of translocation. *Environmental Pollution* **335**, (2023).

135. Freitas, T. B. N., Leite, T. S., de Ramos, B. & Proietti, M. C. Understanding the interactions between cephalopods and marine litter: A research evaluation with identification of gaps and future perspectives. *Mar Pollut Bull* **190**, (2023).

136. Oliveira, A. R. *et al.* Microplastics presence in cultured and wild-caught cuttlefish, Sepia officinalis. *Mar Pollut Bull* **160**, (2020).

137. Galloway, T. S., Cole, M. & Lewis, C. Interactions of microplastic debris throughout the marine ecosystem. *Nat Ecol Evol* **1**, (2017).

138. Sambolino, A. *et al.* Microplastic ingestion and plastic additive detection in pelagic squid and fish: Implications for bioindicators and plastic tracers in open oceanic food webs. *Science of the Total Environment* **894**, (2023).

139. Koelmans, A. A., Bakir, A., Burton, G. A. & Janssen, C. R. Microplastic as a Vector for Chemicals in the Aquatic Environment: Critical Review and Model-Supported Reinterpretation of Empirical Studies. *Environ Sci Technol* **50**, 3315–3326 (2016).

140. Diepens, N. J. & Koelmans, A. A. Accumulation of Plastic Debris and Associated Contaminants in Aquatic Food Webs. *Environ Sci Technol* **52**, 8510–8520 (2018).

141. Goldstein, M. C., Rosenberg, M. & Cheng, L. Increased oceanic microplastic debris enhances oviposition in an endemic pelagic insect. *Biol Lett* **8**, 817–820 (2012).

142. Jang, M. *et al.* Styrofoam Debris as a Source of Hazardous Additives for Marine Organisms. *Environ Sci Technol* **50**, 4951–4960 (2016).

143. Goldstein, M. C. & Goodwin, D. S. Gooseneck barnacles (Lepas spp.) ingest microplastic debris in the north Pacific subtropical gyre. *PeerJ* **2013**, (2013).

144. Richon, C. *et al.* Model exploration of microplastic effects on zooplankton grazing reveal potential impacts on the global carbon cycle. *Environmental Research Letters* (2024) doi:10.1088/1748-9326/ad5195.

Supplementary Figures

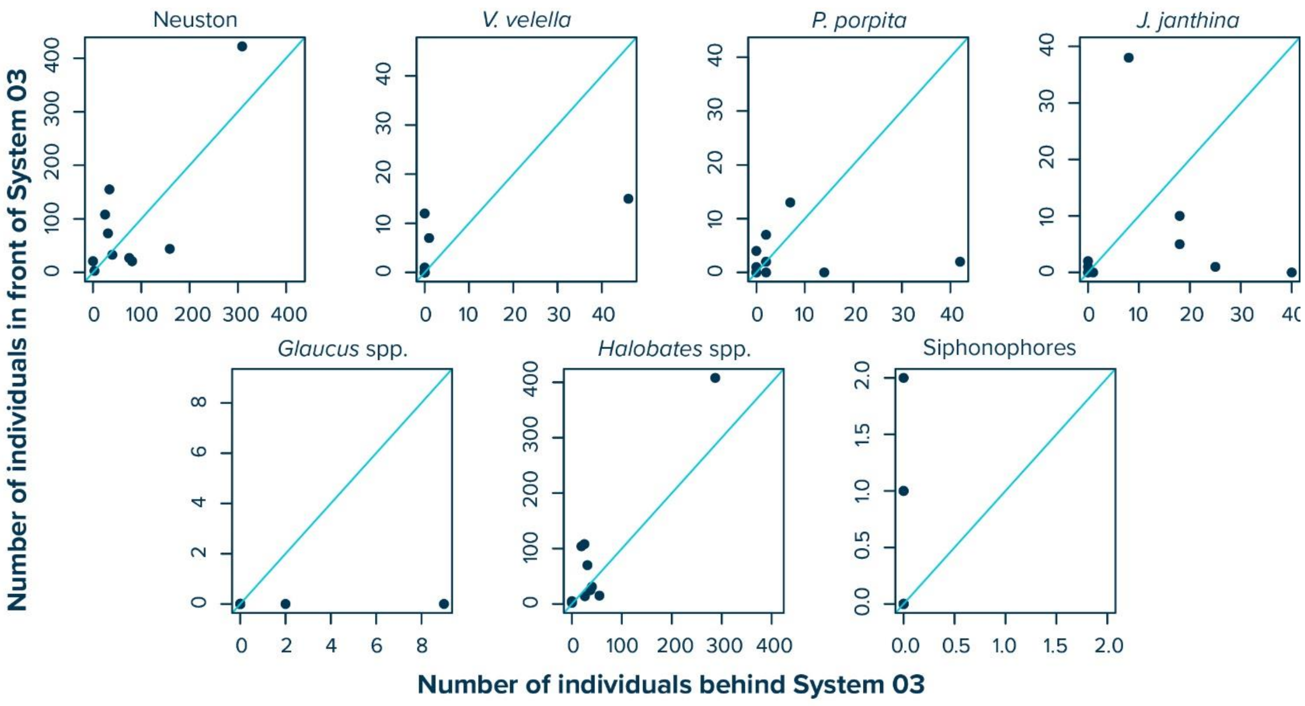

Figure S1 | Comparison of neuston counts (number of individuals) observed in Manta trawl samples concurrently collected from in front (y axis) and from behind (x axis) The Ocean Cleanup's System 03 in 2023. The blue lines indicate a 1:1 ratio, i.e., equal counts in front and behind. Points above the blue line indicate samples that showed higher counts in front of the cleanup system compared to behind, while points below the blue line represent samples with lower counts in front vs behind the cleanup system. For values, the reader is referred to Table S8.

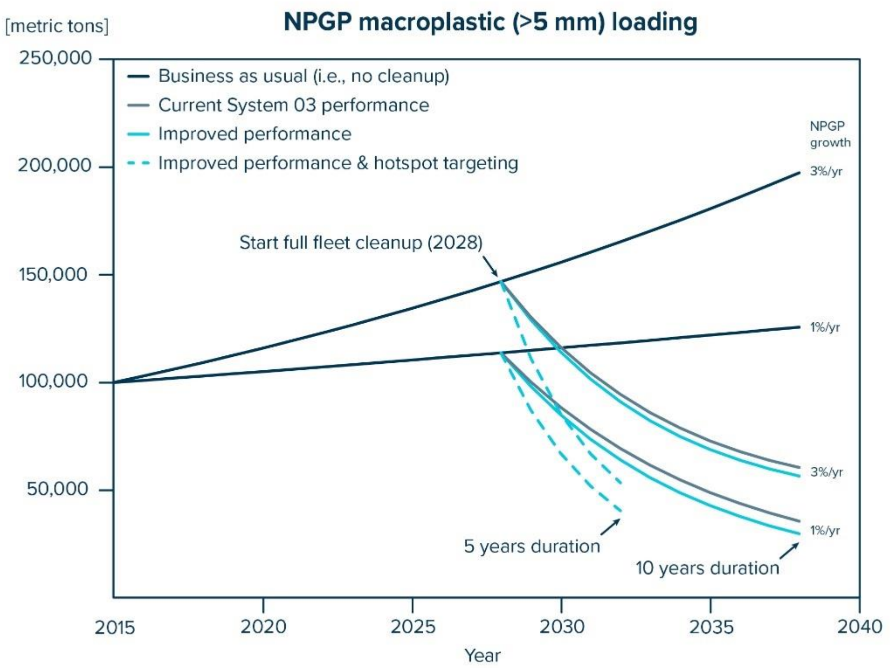

Figure S2 | Predicted plastic mass in the NPGP in metric tons assuming a 1% and 3% annual growth rate of the plastic mass under a business-as-usual scenario (i.e., no cleanup; dark blue), a 10 year cleanup duration and current System 03 performance (gray), a 10 year cleanup duration and improved cleanup performance (light blue), as well as a 5 year cleanup duration and improved hotspot targeting (dashed light blue). Model assumptions are presented in Table S10.

Supplementary Tables

Table S1 | Impact measures used to assess the vulnerability of ecological guilds to plastic pollution and cleanup efforts in the North Pacific Ocean, including within the North Pacific Garbage Patch (NPGP) and beyond. Extent refers to the geographical scale of the impact. Duration is a measure for the persistence of the stressor. Intensity refers to health effects associated with the impact. Reversibility accounts for the capability of an organism to recover from the stressor. Frequency is a measure for the regularity of interactions between the stressor and individual organisms.

Measure	Value	Description
**Extent**	1	NPGP
	2	Beyond NPGP (surface ocean)
	3	Beyond NPGP (surface ocean & deep sea)
**Duration**	1	Short-term (<1 yr)
	2	Mid-term (1-10 yrs)
	3	Long-term (>10 yrs)
**Intensity**	1	Low (mostly sublethal effects)
	2	Moderate (<5% mortality)
	3	High (>5% mortality)
**Reversibility**	1	Reversible
	2	Difficult to reverse
	3	Irreversible
**Frequency**	1	Rare (<5%)
	2	Occasional (6-10%)
	3	Frequent or abundant (>10%)
**Certainty**	1	Low (very little or no empirical work exists)
	2	Moderate (experts have some personal experience)
	3	Medium (some empirical work exists)
	4	High (body of empirical work exists)
	5	Very high (sufficiently proven)

Table S2 | Scoring of impacts of macroplastics (> 5 mm), microplastics (< 5 mm), and plastic cleanup (taking The Ocean Cleanup as a case study) on ecological guilds in the North Pacific Garbage Patch . Values in parentheses represent certainty scores. All scores were derived based on finding consensus among the authors and were supported by literature and additional data where possible (see Table S1 for scoring criteria). Values for vulnerability and uncertainty scores were derived by applying equations (1) and (2), respectively (see methods section in the main manuscript).

Guild	Extent	Duration	Intensity	Reversibility	Frequency	Vulnerability score	Uncertainty score
**Zooplankton**							
Macroplastics	2 (4)	3 (3)	2 (1)	1 (1)	1 (3)	1.64	0.49
Microplastics	3 (4)	3 (3)	2 (3)	2 (1)	3 (3)	2.55	0.39
Cleanup	1 (3)	2 (3)	1 (3)	1 (2)	3 (1)	1.43	0.45
**Neuston**							
Macroplastics	2 (4)	3 (3)	2 (1)	1 (1)	1 (3)	1.64	0.49
Microplastics	3 (4)	3 (3)	1 (1)	2 (1)	3 (3)	2.22	0.49
Cleanup	1 (3)	2 (3)	2 (3)	2 (2)	3 (2)	1.89	0.39
**Bony fish**							
Macroplastics	2 (4)	3 (3)	2 (1)	2 (1)	1 (3)	1.89	0.49
Microplastics	3 (4)	3 (3)	2 (3)	2 (1)	3 (3)	2.55	0.39
Cleanup	1 (3)	2 (3)	3 (5)	3 (5)	2 (2)	2.05	0.29
**Elasmobranchs**							
Macroplastics	2 (4)	3 (3)	2 (1)	2 (1)	3 (1)	2.35	0.61
Microplastics	3 (4)	3 (3)	2 (1)	2 (1)	3 (3)	2.55	0.49
Cleanup	1 (3)	2 (3)	3 (5)	3 (5)	2 (2)	2.05	0.29
**Sea turtles**							
Macroplastics	2 (4)	3 (3)	3 (3)	2 (1)	3 (3)	2.55	0.39
Microplastics	3 (4)	3 (3)	1 (1)	2 (1)	3 (3)	2.22	0.49
Cleanup	1 (3)	2 (3)	3 (3)	2 (2)	3 (3)	2.05	0.36
**Seabirds**							
Macroplastics	2 (4)	3 (3)	3 (4)	3 (3)	3 (3)	2.77	0.30
Microplastics	3 (4)	3 (3)	1 (3)	2 (1)	3 (4)	2.22	0.37
Cleanup	1 (3)	2 (3)	1 (3)	1 (2)	1 (3)	1.15	0.36
**Mammals**							
Macroplastics	2 (4)	3 (3)	2 (3)	2 (1)	3 (1)	2.35	0.49
Microplastics	3 (4)	3 (3)	1 (1)	2 (1)	3 (1)	2.22	0.61
Cleanup	1 (3)	2 (3)	1 (4)	1 (4)	1 (4)	1.15	0.28
**Cephalopods**							
Macroplastics	-	-	-	-	-	1.00	1.00
Microplastics	3 (4)	3 (3)	1 (1)	2 (1)	2 (1)	2.05	0.61
Cleanup	1 (3)	2 (3)	3 (4)	3 (5)	1 (3)	1.78	0.28
**Rafting species**							
Macroplastics	-	-	-	-	-	1.00	0.33
Microplastics	3 (4)	3 (3)	1 (1)	2 (1)	3 (1)	2.22	0.61
Cleanup	1 (3)	2 (3)	3 (5)	3 (5)	3 (4)	2.22	0.26

Table S3 | Average values (± 1 standard deviation) for total vulnerability score, as well as the extent, duration, intensity, reversibility, frequency of impacts and their respective uncertainty scores across all nine ecological guilds considered in this study (i.e., zooplankton, neuston, bony fish, elasmobranchs, sea turtles, seabirds, marine mammals, cephalopods, and rafting species).

	**Macroplastic**		**Microplastics**		**Cleanup**	
	**Mean score**	**Mean uncertainty**	**Mean score**	**Mean uncertainty**	**Mean score**	**Mean uncertainty**
**Vulnerability**	**1.91 ± 0.61**	**0.51 ± 0.20**	**2.31 ± 0.18**	**0.49 ± 0.09**	**1.75 ± 0.38**	**0.33 ± 0.06**
Impact extent	2.00 ± 0.00	0.25 ± 0.00	3.00 ± 0.00	0.25 ± 0.00	1.00 ± 0.00	0.33 ± 0.00
Impact duration	3.00 ± 0.00	0.33 ± 0.00	3.00 ± 0.00	0.33 ± 0.00	2.00 ± 0.00	0.33 ± 0.00
Impact intensity	2.29 ± 0.45	0.50 ± 0.34	1.33 ± 0.47	0.60 ± 0.31	2.22 ± 0.92	0.26 ± 0.06
Impact reversibility	1.86 ± 0.64	0.78 ± 0.23	2.00 ± 0.00	1.00 ± 0.00	2.11 ± 0.87	0.28 ± 0.14
Impact frequency	2.14 ± 0.99	0.41 ± 0.30	2.89 ± 0.31	0.41 ± 0.32	2.11 ± 0.87	0.38 ± 0.22

Table S4 | Total **primary incidental catch** (i.e., organisms that were directly captured by the operations) throughout 18 cleanup campaigns in the NPGP by The Ocean Cleanup between July 2021 and November 2023. The lowest taxonomic levels identified are indicated in parentheses. Percentages for the animal group (bold) are in relation to the total catch; percentages for lower taxonomic levels (not bold) are in relation to the total catch within the respective animal group.

	IUCN status*	Habitat**	Amount		Weight	
			[#]	[%]	[kg]	[%]
**Cephalopods**			**70**	**0.3**	**45.1**	**2.7**
Squid (Decapodiformes)	Unidentified	Unidentified	41	58.6	27.7	61.5
Octopus (Octopoda)	Unidentified	Unidentified	22	31.4	12.2	27.2
Argonaut (*Argonauta* spp.)	Least concern	Oceanic, pelagic	5	7.1	1.9	4.2
Unknown cephalopod	Unidentified	Unidentified	2	2.9	3.2	7.1
**Cnidarians**			**1,000**	**3.9**	**1.0**	**0.1**
By-the-wind sailor (*Velella velella*)	Not evaluated	Neustonic	1,000	100.0	1.0	100.0
**Elasmobranchs**			**3,031**	**11.8**	**457.9**	**27.3**
Pygmy/cookiecutter shark (Dalatiidae)	Least concern	Oceanic, several depths	2,983	98.4	185.9	40.6
Blue shark *(Prionace glauca)*	Near threatened	Oceanic, epipelagic	43	1.4	271.3	59.2
Kitefin shark (*Dalatias licha*)	Vulnerable	Oceanic, epibenthic	3	0.1	0.2	0.03
Pelagic stingray (*Pteroplatytrygon violacea*)	Least concern	Oceanic, epipelagic	2	0.1	0.6	0.1
**Bony fish**			**21,649**	**84.0**	**734.4**	**43.9**
Blennies (Blenniidae, multiple species)	Unidentified	Coastal (commonly reef-associated). Can be associated with floating debris	9,397	43.4	41.8	5.7
Indo-Pacific Sergeant (*Abudefduf vaigiensis*)	Least concern	Coastal (commonly reef-associated). Can be associated with floating debris	7,050	32.6	148.7	20.3
Flying Fish (*Hirundichthys* spp.)	Least concern	Oceanic, epipelagic	2,297	10.6	203.1	27.7
Amberjack (*Seriola* spp.)	Least concern	Coastal/oceanic, benthopelagic.	1,475	6.8	116.7	15.9
Freckled driftfish (*Psenes cyanophrys*)	Least concern	Oceanic, epipelagic. Can be associated with floating debris	392	1.8	12.9	1.8
Sargassumfish (*Histrio histrio*)	Least concern	Coastal. Associated with floating debris	360	1.7	22.8	3.1
Garfish (*Hemiramphus* sp.)	Least concern	Coastal/oceanic, epipelagic	183	0.9	6.7	0.9
Lanternfish (Myctophiformes)	Least concern	Oceanic, mesopelagic	151	0.7	17.4	2.4
Filefish (*Aluterus* sp.)	Least concern	Coastal (commonly reef-associated). Can be associated with floating debris	47	0.2	35.8	4.9
Common dolphinfish (*Coryphaena hippurus*)	Least concern	Coastal/oceanic, epipelagic	45	0.2	12.4	1.7
Porcupinefish (*Diodon* sp.)	Least concern	Coastal (commonly reef-associated).	41	0.2	42.6	5.8
Spotted knifejaw (*Oplegnathus punctatus*)	Not evaluated	Coastal (commonly reef-associated). Can be associated with drifting debris	29	0.1	35.7	4.9
Rough triggerfish (*Canthidermis maculata*)	Least concern	Coastal/oceanic, epipelagic. Can be associated with floating debris	29	0.1	10.2	1.4
Pacific pomfret (*Brama japonica*)	Not evaluated	Oceanic, epipelagic	11	0.1	11.6	1.6
Pilotfish (*Naucrates ductor*)	Least concern	Oceanic, epi/mesopelagic. Can be associated with floating debris	7	0.03	0.2	0.02
Sea chub (Kyphosidae)	Least concern	Coastal (commonly reef-associated). Can be associated with floating debris	6	0.03	1.8	0.2
Shortbill spearfish (*Tetrapturus angustirostris*)	Data deficient	Oceanic, epipelagic	5	0.02	5.4	0.7
Ocean sunfish (*Mola mola*)	Vulnerable	Oceanic, epi/mesopelagic	3	0.01	0.1	0.01
Bigeye cigarfish (*Cubiceps pauciradiatus*)	Least concern	Oceanic, epipelagic	2	0.01	0.02	0.003
Striped beakfish (*Oplegnathus fasciatus*)	Not evaluated	Coastal (commonly reef-associated). Can be associated with floating debris	2	0.01	1.0	0.1
Mackerel (Gempylidae)	Least concern	Oceanic, epipelagic	1	0.01	0.4	0.1
Remora (*Remora* sp.)	Least concern	Associated with larger organisms	1	0.01	0.5	0.1
Unknown bony fish	Unidentified	Unidentified	115	0.5	6.8	0.9
**Sea turtles**			**21**	**0.1**	**149.8**	**8.9**
Loggerhead (*Caretta caretta*)	Vulnerable	Coastal/Oceanic, mostly epipelagic	14	66.7	116.9	78.1
Green (*Chelonia mydas*)	Endangered	Coastal/Oceanic, mostly epipelagic	5	23.8	14.1	9.4
Olive ridley (*Lepidochelys olivacea*)	Vulnerable	Neritic/Oceanic, mostly epipelagic	2	9.5	18.8	12.5
**Unidentified*****	Unidentified	Unidentified	n/a	n/a	**286.4**	**17.1**
**Total**			**25,771**		**1674.6**	**0.5******

*International Union for the Conservation of Nature (IUCN) Red List status (global) checked on 10 April 2024. **Most common habitat of the mature life stage of species/groups. ***Unidentifiable biological matter remaining after processing of plastic catch on deck (mostly consisting of fish remains). ****Percentage of total primary incidental catch (1,674.6 kg) vs total waste collected for which incidental catch analysis was done (341,724 kg).

Table S5 | Total **secondary incidental catch** (i.e., organisms that live on or attached to the plastic, or have become entangled in it prior to entering the cleanup system as identified based on onboard observers) throughout 18 cleanup campaigns in the NPGP by The Ocean Cleanup between July 2021 and November 2023. The lowest taxonomic levels identified are indicated in parentheses. Percentages for the animal group (bold) are in relation to the total catch; percentages for lower taxonomic levels (not bold) are in relation to the total catch within the respective animal group. Note that unlike data collected for primary incidental catch (Table S4), numbers on secondary bycatch are more uncertain due to logistical difficulties associated with quantifying organisms attached to the plastic and likely loss of some sessile organisms during plastic extraction. Thus, these numbers are likely underestimating true impacts.

	**IUCN status***	**Habitat****	**Amount**		**Weight**	
			**[#]**	**[%]**	**[kg]**	**[%]**
**Sea worms**			**5**	**0.01**	**0.5**	**0.4**
Annelida	Unidentified	Benthic	5	100	0.5	100
**Cnidarians**			**220**	**0.4**	**2.8**	**2.1**
Anemone (Actiniaria)	Unidentified	Benthic, mostly sessile	215	97.7	2.2	77.2
Coral (Scleractinia)	Unidentified	Benthic, sessile	1	0.5	0.04	1.5
Zoanthid (Zoantharia)	Unidentified	Benthic, sessile	1	0.5	0.02	0.7
Unknown cnidarian	Unidentified	Unidentified	3	1.4	0.6	1.4
**Crustaceans**			**48,509**	**98.8**	**107.1**	**81.3**
Crab (Brachyura, multiple species)	Unidentified	Coastal/oceanic, primarily benthic or rafting. Commonly associated with floating debris	24,283	50.1	53.7	50.2
Barnacle (Cirripedia, multiple species)	Unidentified	Coastal/oceanic, sessile. Commonly associated with floating debris	24,225	49.9	53.3	49.7
Amphipod (Amphipoda)	Unidentified	All marine habitats	1	0.002	0.1	0.1
**Echinoderms**			**12**	**0.02**	**0.1**	**0.1**
Brittle star (Ophiuroidea)	Unidentified	Benthic	12	100	0.1	100
**Bryozoans**			**300**	**0.6**	**0.9**	**0.7**
Bryozoa	Unidentified	Benthic	300	100	0.9	100
**Mollusks**			**39**	**0.1**	**1.1**	**0.8**
Bivalve (Bivalvia)	Unidentified	Benthic	38	97.4	1.1	99.5
Gastropod (Gastropoda)	Unidentified	Benthic	1	2.6	0.01	0.5
**Sponges**			**7**	**0.01**	**0.1**	**0.1**
Porifera	Unidentified	Benthic, sessile	7	100	0.1	100
**Sea turtles**			**2**	**0.004**	**19**	**14.4**
Green turtle (*Chelonia mydas*)	Endangered	Coastal/oceanic, mostly epipelagic	1	50	4	21.1
Loggerhead turtle (*Caretta caretta*)	Vulnerable	Coastal/oceanic, mostly epipelagic	1	50	15	78.9
**Total**			**49,094**		**131.7**	**0.04*****

*International Union for the Conservation of Nature (IUCN) Red List status (global) checked on 10 April 2024. **Most common habitat of the mature life stage of species/groups. ***Percentage of secondary incidental catch (131.7 kg) vs total waste collected for which incidental catch analysis was done (341,724 kg).

Table S6 | Total tertiary incidental catch (i.e., remains of organisms that died prior to encountering the cleanup system) throughout 18 cleanup campaigns in the NPGP by The Ocean Cleanup between July 2021 and November 2023. The lowest taxonomic levels identified are indicated in parentheses. Percentages for the animal group (bold) are in relation to the total catch; percentages for lower taxonomic levels (not bold) are in relation to the total catch within the respective animal group.

	**IUCN status***	**Habitat**	**Amount**		**Weight**	
			**[#]**	**[%]**	**[kg]**	**[%]**
**Seabirds**			**7**	**41.2**	**5.8**	**17.9**
Northern fulmar (*Fulmarus glacialis*)	Least concern	Pelagic	2	28.6	1.5	25.7
Storm petrel (*Hydrobates* sp.)	n/a	Pelagic	2	28.6	0.3	5.8
Albatross (Diomedeidae)	n/a	Pelagic	1	14.3	2.9	49.7
Sooty shearwater (*Ardenna grisea*)	Near threatened	Pelagic	1	14.3	0.7	12.0
Unknown seabird carcass	n/a	n/a	1	14.3	0.4	6.8
**Sea turtles**			**10**	**58.8**	**26.7**	**82.1**
Loggerhead turtle (*Caretta caretta*)	Vulnerable	Neritic/Oceanic, mostly epipelagic	5	50	15.4	57.6
Green turtle (*Chelonia mydas*)	Endangered	Neritic/Oceanic, mostly epipelagic	4	40	10.8	40.5
Unknown turtle carcass (Cheloniidae)	n/a	Neritic/Oceanic, mostly epipelagic	1	10	0.5	1.9
**Total**			**17**		**32.5**	**0.01****

*International Union for the Conservation of Nature (IUCN) Red List status (global) checked on 10 April 2024. ****Percentage of tertiary incidental catch (32.5 kg) vs total waste collected for which incidental catch analysis was done (341,724 kg).

Table S7 | Total marine life observations by onboard protected species observers throughout 18 cleanup campaigns in the NPGP by The Ocean Cleanup between July 2021 and November 2023. Observations were done both during transit between the NPGP and Victoria (CAN), as well as during cleanup operations in the NPGP. Total number of sightings are reported, as well as number of animals sighted per 100 hours of observations to account for different observational efforts during transit (3,972 hours) vs operations (7,837 hours). The lowest taxonomic levels identified are indicated in parentheses.

		IUCN status*	# animals		#animals/100h	
			**Transit**	**Operations**	**Transit**	**Operations**
**Bony fish**			**27**	**5**	**0.68**	**0.06**
	Ocean sunfish (*Mola mola*)	Vulnerable	25	5	0.63	0.06
	Unidentified billfish	NA	2	0	0.05	0.00
**Elasmobranch**			**4**	**60**	**0.10**	**0.77**
	Blue shark (*Prionace glauca*)	Near threatened	0	57	0.00	0.69
	White shark (*Carcharodon carcharias*)	Vulnerable	1	0	0.03	0.00
	Unidentified shark	NA	3	1	0.08	0.01
	Pelagic stingray (*Pteroplatytrygon violacea*)	Least concern	0	2	0.00	0.03
**Marine mammals**			**2,901**	**107**	**73.03**	**1.37**
Mysticete			*641*	*27*	*16.15*	*0.34*
	Blue whale (*Balaenoptera musculus*)	Endangered	2	2	0.05	0.03
	Fin whale (*Balaenoptera physalus*)	Vulnerable	38	2	0.96	0.03
	Gray whale (*Eschrichtius robustus*)	Least concern	1	0	0.03	0
	Humpback whale (*Megaptera novaeangliae*)	Least concern	500	5	12.59	0.06
	Minke whale (*Balaenoptera acutorostrata*)	Least concern	1	1	0.03	0.01
	Sei whale (*Balaenoptera borealis*)	Endangered	5	1	0.13	0.01
	Unidentified Balaenopteridae	NA	43	11	1.08	0.14
	Unidentified Mysticeti	NA	51	5	1.28	0.06
Odontocete			*2,187*	*76*	*55.84*	*1.03*
	Blainville's beaked whale (*Mesoplodon densirostris*)	Least concern	15	0	0.38	0.00
	Dall's porpoise (*Phocoenoides dalli*)	Least concern	37	0	0.93	0.00
	False killer whale (*Pseudorca crassidens*)	Near threatened	0	12	0.00	0.15
	Harbour porpoise (*Phocoena phocoena*)	Least concern	39	0	0.98	0.00
	Killer whale (*Orcinus orca*)	Data deficient	60	0	1.51	0.00
	Northern right whale dolphin (*Lissodelphis borealis*)	Least concern	161	0	4.05	0.00
	Pacific white-sided dolphin (*Lagenorhynchus obliquidens*)	Least concern	180	0	4.53	0.00
	Risso's dolphin (*Grampus griseus*)	Least concern	19	0	0.48	0.00
	Short-beaked common dolphin (*Delphinus delphis*)	Least concern	1,345	0	33.86	0.00
	Short-finned pilot whale (*Globicephala macrorhynchus*)	Least concern	0	49	0.00	0.63
	Sperm whale (*Physeter macrocephalus*)	Vulnerable	44	3	1.11	0.04
	Unidentified Ziphiidae	NA	3	6	0.08	0.08
	Unidentified Delphinidae	NA	208	6	5.24	0.08
	Unidentified Delphinidae/Phocoenidae	NA	12	0	0.30	0.00
	Unidentified Odontocete	NA	64	0	1.61	0.00
Unidentified Cetacean		NA	7	4	0.18	0.05
Unidentified whale		NA	24	0	0.60	0.00
Pinniped			*42*	*0*	*1.06*	*0*
	California sea lion (*Zalophus californianus*)	Least concern	8	0	0.20	0.00
	Northern fur seal (*Callorhinus ursinus*)	Vulnerable	7	0	0.18	0.00
	Steller sea lion (*Eumetopias jubatus*)	Near threatened	8	0	0.20	0.00
	Unidentified pinniped	NA	19	0	0.48	0.00
**Sea turtle**			**8**	**68**	**0.20**	**0.87**
	Green turtle (*Chelonia mydas*)	Endangered	0	15	0	0.19
	Loggerhead turtle (*Caretta caretta*)	Vulnerable	2	31	0.05	0.40
	Olive ridley turtle (*Lepidochelys olivacea*)	Vulnerable	0	3	0	0.04
	Unidentified turtle (Cheloniidae)	NA	6	19	0.15	0.24
**Birds**			**2,205**	**1,055**	**55.51**	**13.46**
	Arctic Tern (*Sterna paradisaea*)	Least concern	1	0	0.03	0
	Black Turnstone (*Arenaria melanocephala*)	Least concern	1	0	0.03	0
	Black-Footed Albatross (*Phoebastria nigripes*)	Near threatened	94	149	2.37	1.9
	Black-legged Kittiwake (*Rissa tridactyla*)	Vulnerable	1	0	0.03	0
	Black-winged Petrel (*Pterodroma nigripennis*)	Least concern	0	1	0	0.01

	IUCN status*	# animals		#animals/100h	
Bonaparte's Gull (*Larus philadelphia*)	Least concern	1	0	0.03	0
		Transit	Operations	Transit	Operations
Brown Booby (*Sula leucogaster*)	Least concern	5	21	0.13	0.27
Buller's Shearwater (*Ardenna bulleri*)	Vulnerable	5	1	0.13	0.01
California Gull (*Larus californicus*)	Least concern	1	0	0.03	0
Canada Goose (*Branta canadensis*)	Least concern	21	0	0.53	0
Cassin's Auklet (*Ptychoramphus aleuticus*)	Near threatened	29	0	0.73	0
Common Loon (*Gavia immer*)	Least concern	3	0	0.08	0
Common Murre (*Uria aalge*)	Least concern	159	0	4	0
Cook's Petrel (*Pterodroma cookii*)	Vulnerable	0	1	0	0.01
Double-crested Cormorant (*Nannopterum auritum*)	Least concern	1	0	0.03	0
Fork-tailed Storm Petrel (*Hydrobates furcatus*)	Least concern	4	1	0.10	0.01
Golden-crowned Kinglet (*Regulus satrapa*)	Least concern	1	0	0.03	0
Herring Gull (*Larus* sp.)	Least concern	7	0	0.18	0
Laysan Albatross (*Phoebastria immutabilis*)	Near threatened	65	644	1.64	8.22
Leach's Storm Petrel (*Hydrobates leucorhous*)	Vulnerable	119	112	3	1.43
Masked Booby (*Sula dactylatra*)	Least concern	1	20	0.03	0.26
Northern Fulmar (*Fulmarus glacialis*)	Least concern	23	1	0.58	0.01
Pacific Golden Plover (*Pluvialis fulva*)	Least concern	2	27	0.05	0.34
Parasitic Jaeger (*Stercorarius parasiticus*)	Least concern	10	0	0.25	0
Peregrine Falcon (*Falco peregrinus*)	Least concern	2	3	0.05	0.04
Pigeon Guillemot (*Cepphus columba*)	Least concern	9	0	0.23	0
Pomarine Jaeger (*Stercorarius pomarinus*)	Least concern	2	0	0.05	0
Red Phalarope (*Phalaropus fulicarius*)	Least concern	459	0	11.55	0
Red-Footed Booby (*Sula sula*)	Least concern	1	20	0.03	0.26
Red-Tailed Tropicbird (*Phaethon rubricauda*)	Least concern	8	24	0.2	0.31
Rhinoceros Auklet (*Cerorhinca monocerata*)	Least concern	3	0	0.08	0
Savannah Sparrow (*Passerculus sandwichensis*)	Least concern	3	1	0.08	0.01
Sooty Shearwater (*Ardenna grisea*)	Near threatened	175	8	4.41	0.1
Townsend's Warbler (*Setophaga townsendi*)	Least concern	1	0	0.03	0
Tufted Puffin (*Fratercula cirrhata*)	Least concern	3	0	0.08	0
Yellow Warbler (*Setophaga petechia*)	Least concern	21	0	0.53	0
Unidentified Alcid (Alcidae)	NA	5	0	0.13	0
Unidentified Booby (*Sula* sp.)	NA	0	1	0	0.01
Unidentified Duck (Anatidae)	NA	170	0	4.28	0
Unidentified Gull (Laridae)	NA	660	5	16.61	0.06
Unidentified Passerine (Passeriforme)	NA	5	0	0.13	0
Unidentified Petrel (*Pterodroma* sp.)	NA	2	0	0.05	0
Unidentified Phalarope (Scolopacidae)	Least concern	1	0	0.03	0
Unidentified Sandpiper (Scolopacidae)	NA	16	0	0.4	0
Unidentified Shearwater (Procellariidae)	NA	50	3	1.26	0.04
Unidentified Storm Petrel (*Hydrobates* sp.)	NA	4	3	0.10	0.04
Unidentified Tern (Sterninae)	NA	13	0	0.33	0
Unidentified bird	NA	38	9	0.96	0.11
**Total**		**5,145**	**1,295**	**129.52**	**16.52**

*International Union for the Conservation of Nature (IUCN) Red List status (global) checked on 10 April 2024.

Table S8 | Manta trawl samples concurrently collected in front and behind The Ocean Cleanup's System 03 in 2023 to evaluate cleanup impacts on obligate neuston.

**Location**	**Date**	**Start time**	**End time**	**Sea state** *[BF]*	**Velella** *[#]*	**Porpita** *[#]*	**Janthina** *[#]*	**Glaucus** *[#]*	**Halobates** *[#]*	**Siphonophores** *[#]*	**Neuston** *[#]*
Front	31.07.	16:00	16:30	1	7	0	0	0	14	0	21
Front	31.07.	16:40	17:10	1	15	2	1	0	25	1	44
Behind	31.07.	15:59	16:29	1	1	14	40	0	26	0	81
Behind	31.07.	16:40	17:10	1	46	42	25	9	37	0	159
Front	09.09.	09:58	10:28	3	0	0	1	0	31	1	33
Front	09.09.	10:38	11:08	3	0	0	0	0	108	0	108
Behind	09.09.	09:58	10:28	3	0	0	0	0	40	0	40
Behind	09.09.	10:38	11:08	3	0	0	0	0	25	0	25
Front	17.09.	10:01	10:31	2	0	1	2	0	70	0	73
Front	17.09.	10:38	11:08	2	0	2	10	0	408	2	422
Behind	17.09.	10:01	10:31	2	0	0	0	0	31	0	31
Behind	17.09.	10:38	11:08	2	0	2	18	2	287	0	309
Front	23.10.	13:47	14:17	3	0	13	38	0	104	0	155
Front	23.10.	14:25	14:55	3	0	7	5	0	15	0	27
Behind	23.10.	13:47	14:17	3	0	7	8	0	19	0	34
Behind	23.10.	14:25	14:55	3	0	2	18	0	55	0	75
Front	03.11.	15:05	15:35	4	1	0	0	0	2	0	3
Front	03.11.	15:42	16:12	4	12	4	0	0	5	0	21
Behind	03.11.	15:05	15:35	4	0	2	1	0	0	0	3
Behind	03.11.	15:42	16:12	4	0	0	0	0	0	0	0

Table S9 | Overview of assumptions made for different predictions in future macro- and microplastic concentrations in the NPGP.

Macroplastics (> 5 mm)	High degradation scenario	Low degradation scenario	References
Concentrations in 2015 [kg/km^2]	67 (48 – 89)	67 (48 – 89)	[1]
Increase of macroplastics afloat in the global surface ocean	4.0%/yr	4.0%/yr	[20]
Degradation of macroplastics into particles < 5 mm	3.0%/yr	1.0%/yr	[10,20]
Net mass growth rate (increase – degradation)	1.0%/yr	3.0%/yr	

Microplastics (500 µm – 5 mm)	74% external inputs	96% external inputs	
Concentrations in 2015 [#/km^2]	678,374<br>(422,288 – 1,360,431)	678,374<br>(422,288 – 1,360,431)	[1]
Observed net growth rate between 2015 to 2022	6.00%/yr	6.00%/yr	[2]
Annual growth due to degradation of macroplastics	1.56%	0.24%	
Annual growth due to external inputs	4.44%	5.76%	
Net annual growth rate with no cleanup of macroplastics	6.00%	6.00%	
Net growth rate with 50% cleanup of macroplastics	5.22%	5.88%	
Net growth rate with 70% cleanup of macroplastics	4.91%	5.83%	
Net growth rate with 80% cleanup of macroplastics	4.75%	5.81%	
Net growth rate with 90% cleanup of macroplastics	4.60%	5.78%	

Table S10 | Overview of assumptions made to estimate carbon emissions needed to reach an 80% reduction in macroplastic (>5 mm) loading in the NPGP compared to business-as-usual (i.e., no cleanup). The predicted NPGP mass load for each scenario are shown in Figure S2. Note that the model and associated calculations are provided in the Supplementary Material.

	**Scenario 1 (current performance)**		**Scenario 2 (improved performance)**		**Scenario 3 (hotspot targeting)**	
	**High**	**Low**	**High**	**Low**	**High**	**Low**
Plastic mass load in 2015 [tons]	100,000	100,000	100,000	100,000	100,000	100,000
**Net mass growth rate [%/yr]**	**3**	**1**	**3**	**1**	**3**	**1**
Effective span (cleanup system) [m]	1050	1050	1350	1350	1350	1350
Speed through water [m/s]	0.65	0.65	0.8	0.8	0.8	0.8
Swept area [km^2/h]	2.46	2.46	3.89	3.89	3.89	3.89
Towing uptime [%]	45	48	71	72	72	72
Relative encountered density [%]	100	100	100	100	210	210
Retention efficiency [%]	47	42	47	42	47	42
**# systems in operation**	**53**	**54**	**20**	**20**	**18**	**18**
# of vessels	106	108	44	44	39	39
**Years of operations**	**10**	**10**	**10**	**10**	**5**	**5**
Total swept area [km^2/yr]	513,331	557,884	483,636	490,448	441,403	441,403
Time spent in port [%]	4	4	2	2	2	2
Time spent in transit [%]	25	22	9	9	9	9
Time spent in-field operations [%]	26	26	18	16	16	16
Time spent towing [%]	45	48	71	72	72	72
**C emissions full fleet [kilotons/yr)**	**285**	**290**	**96**	**96**	**85**	**85**
**C emissions cumulative [kilotons]**	**2,847**	**2,901**	**961**	**963**	**427**	**427**

Table S11 | Microplastics impacts on ocean biogeochemical processes, estimated by extracting results from a global coupled physical-biogeochemical model [144] for the NPGP (defined as surface waters in the North Pacific subtropical gyre containing more than 1 kg/km^2 of microplastics [1]). Impacts were modeled by using a microplastic to prey ratio (ɑ) of 0.9 (high threshold, and thus low impacts) and 0.1 (low threshold and thus high impacts). Integrated impacts in the first 100 m of the NPGP water column were calculated on short (year 10) and long (year 100) timescales. All values are in megaton carbon per year (Mt C/yr).

[Mt C/yr]	**Control**		**Low impacts (ɑ= 0.9)**		**High impacts (ɑ= 0.1)**	
	Year 10	Year 100	Year 10	Year 100	Year 10	Year 100
Primary production	120	330	90	240	60	150
Remineralization	45	125	30	70	10	30
Zooplankton grazing	130	350	80	230	50	130
Carbon export	20	50	13	35	7	20

Table S12 | List of peer-reviewed scientific publications with substantial contributions of data collected during operations by The Ocean Cleanup in the North Pacific Garbage Patch. So far (i.e., by February 2025), operations by The Ocean Cleanup enabled 28 peer-reviewed publications.

Authors	Title	Journal
Kunz et al. (2024)	Transient attracting profiles in the Great Pacific Garbage Patch	Ocean Science
Lebreton et al. (2024)	Seven years into the North Pacific Garbage Patch: legacy plastic fragments rising disproportionally faster than larger floating objects.	Environmental Research Letters
Souza dos Passos et al. (2024)	Hydrothermal liquefaction of plastic marine debris from the North Pacific Garbage Patch	Resources, Conservation & Recycling
Egger et al. (2024)	Densities of neuston often not elevated within plastic hotspots territory inside the North Pacific Garbage Patch	Environmental Research: Ecology
Royer et al. (2024)	Computer vision segmentation model – deep learning for categorizing microplastic debris	Frontiers in Environmental Science
Vaksmaa et al. (2024)	Biodegradation of polyethylene by the marine fungus Parengyodontium album	Science of The Total Environment
De Vries et al. (2023)	Hyperspectral reflectance of pristine, ocean weathered and biofouled plastics from a dry to wet and submerged state	Earth System Science Data
Jimenez et al. (2023)	On the digital Twin of The Ocean Cleanup Systems—Part I: Calibration of the drag coefficients of a netted screen in OrcaFlex using CFD and full-scale experiments	Journal of Marine Science and Engineering
Haram et al. (2023)	Extent and reproduction of coastal species on plastic debris in the North Pacific Subtropical Gyre	Nature Ecology & Evolution
Zhao et al. (2023)	Pelagic microplastics in the North Pacific subtropical gyre: a prevalent anthropogenic component of the particulate organic carbon pool	PNAS Nexus
Delre et al. (2023)	Plastic Photodegradation Under Simulated Marine Conditions	Marine Pollution Bulletin
Sainte-Rose et al. (2023)	Persistency and surface convergence evidenced by two maker buoys in the Great Pacific Garbage Patch	Journal of Marine Science and Engineering
Lebreton (2022)	The status and fate of oceanic garbage patches	Nature Reviews, Earth and Environment
Lebreton et al. (2022)	Industrialised fishing nations largely contribute to floating plastic pollution in the North Pacific subtropical gyre	Scientific Reports
Vaksmaa et al. (2022)	Microbial communities on plastic particles in surface waters differ from subsurface waters of the North Pacific subtropical gyre	Marine Pollution Bulletin
Park et al. (2021)	Detecting the Great Pacific Garbage Patch floating plastic litter using WorldView-3 satellite imagery	Optics Express
De Vries et al. (2021)	Quantifying floating plastic debris at sea using vessel-based optical data and artificial intelligence	Remote Sensing
Peytavin et al. (2021)	Ocean Plastic Assimilator v0.2: assimilation of plastic concentration data into Lagrangian dispersion models	Geoscientific Model Development
Egger et al. (2021)	Relative abundance of floating plastic debris and neuston in the eastern North Pacific Ocean	Frontiers in Marine Science
Garaba et al. (2021)	Concentration, anisotropic and apparent colour effects on optical reflectance properties of virgin and ocean-harvested plastics	Journal of Hazardous Materials
Egger et al. (2020)	A spatially variable scarcity of floating microplastics in the eastern North Pacific Ocean	Environmental Research Letters
Egger et al. (2020)	First evidence of plastic fallout from the North Pacific Garbage Patch	Scientific Reports
Lebreton et al. (2019)	A global mass budget for positively buoyant macroplastic debris in the ocean	Scientific Reports
Gibbs et al. (2019)	Cetacean Sightings within the Great Pacific Garbage Patch	Marine Biodiversity
Garaba et al. (2018)	Sensing ocean plastics with an airborne hyperspectral shortwave IR imager	Environmental Science & Technology
Lebreton et al. (2018)	Evidence that the Great Pacific Garbage Patch is rapidly accumulating plastic	Scientific Reports
Chen et al. (2017)	Pollutants in plastics within the North Pacific subtropical gyre	Environmental Science & Technology
Debeljak et al. (2017)	Extracting DNA from ocean microplastics: a method comparison study	Analytical Methods
